# Co- but not Sequential Infection of DCs Boosts Their HIV-Specific CTL-Stimulatory Capacity

**DOI:** 10.3389/fimmu.2019.01123

**Published:** 2019-05-24

**Authors:** Manuela Schönfeld, Ulla Knackmuss, Parul Chandorkar, Paul Hörtnagl, Thomas John Hope, Arnaud Moris, Rosa Bellmann-Weiler, Cornelia Lass-Flörl, Wilfried Posch, Doris Wilflingseder

**Affiliations:** ^1^Division of Hygiene and Medical Microbiology, Medical University of Innsbruck, Innsbruck, Austria; ^2^Central Institute for Blood Transfusion and Immunological Department, Medical University of Innsbruck, Innsbruck, Austria; ^3^Department of Cell and Molecular Biology, Feinberg School of Medicine, Northwestern University, Chicago, IL, United States; ^4^Sorbonne Université, INSERM, CNRS, Center for Immunology and Microbial Infections - CIMI-Paris, Paris, France; ^5^Institute for Integrative Biology of the Cell (I2BC), CEA, CNRS, Université Paris-Sud, Université Paris-Saclay, Gif-sur-Yvette, France; ^6^Department of Internal Medicine II, Medical University of Innsbruck, Innsbruck, Austria

**Keywords:** HIV-1, STIs, dendritic cell, complement, CTL

## Abstract

Pathogenic bacteria and their microbial products activate dendritic cells (DCs) at mucosal surfaces during sexually transmitted infections (STIs) and therefore might also differently shape DC functions during co-infection with HIV-1. We recently illustrated that complement (C) coating of HIV-1 (HIV-C), as primarily found during the acute phase of infection before appearance of HIV-specific antibodies, by-passed SAMHD1-mediated restriction in DCs and therefore mediated an increased DC activation and antiviral capacity. To determine whether the superior antiviral effects of HIV-C-exposed DCs also apply during STIs, we developed a co-infection model in which DCs were infected with *Chlamydia spp*. simultaneously (HIV-C/Chlam-DCs or HIV/Chlam-DCs) or a sequential infection model, where DCs were exposed to *Chlamydia* for 3 or 24 h (Chlam-DCs) followed by HIV-1 infection. Co-infection of DCs with HIV-1 and *Chlamydia* significantly boosted the CTL-stimulatory capacity compared to HIV-1-loaded iDCs and this boost was independent on the opsonization pattern. This effect was lost in the sequential infection model, when opsonized HIV-1 was added delayed to *Chlamydia*-loaded DCs. The reduction in the CTL-stimulatory capacity of Chlam-DCs was not due to lower HIV-1 binding or infection compared to iDCs or HIV-C/Chlam-DCs, but due to altered fusion and internalization mechanisms within DCs. The CTL-stimulatory capacity of HIV-C in Chlam-DCs correlated with significantly reduced viral fusion compared to iDCs and HIV-C/Chlam-DCs and illustrated considerably increased numbers of HIV-C-containing vacuoles than iDCs. The data indicate that *Chlamydia* co-infection of DCs mediates a transient boost of their HIV-specific CTL-stimulatory and antiviral capacity, while in the sequential infection model this is reversed and associated with hazard to the host.

## Introduction

Dendritic cells (DCs) play a pivotal role in the defense against invading pathogens. DCs reside in the peripheral tissue, where they capture antigens and transport them to lymph nodes to present them to naive T cells. Hence, DCs play a key role in shaping the adaptive immune response. Of all new HIV-1 infections, 60–90 % are caused by sexual transmission (1, 2). Since HIV-1 transmission occurs at mucosal surfaces, DCs are amongst the first cells to encounter the virus (3). At the same time, HIV-1 spontaneously activates the classical complement (C) pathway (4), even in seminal fluid (5), through direct binding of C1q to the viral surface. Therefore, C-opsonized HIV (HIV-C) is accumulating at mucosal sites during early HIV-1 infection (6, 7).

We have previously shown that HIV-C interacts with complement receptors 3 (CR3) and 4 (CR4) on iDCs, whereas non-opsonized HIV binds DCs via gp120 to DC-SIGN (8) and via CD169 (Siglec-1) binding to virions. Furthermore, iDCs were efficiently infected with HIV-C compared to non- or antibody-opsonized HIV (7, 9). HIV-C was able to bypass SAMHD1 restriction in DCs, an intrinsic cellular defense mechanism, which usually inhibits HIV-1 replication in myeloid cells. Thus, complement opsonization of the virus counteracted viral defenses in DCs. DCs exposed to HIV-C had a significantly higher maturation and co-stimulatory capacity compared to DCs exposed to non-opsonized HIV (9).

In general, efficiency of HIV-1 transmission is low (10). However, it is known that viral and bacterial genital infections that cause inflammation or ulcers increase risk of infection and/or susceptibility to HIV transmission (10). Epidemiological studies also revealed a link between an increased incidence of STIs with increased efficiency to transmit the virus to an uninfected partner (11). Among the STIs most commonly associated with high genital HIV loads are *Gardnerella vaginalis* (12, 13) associated with bacterial vaginosis (BV), herpes simplex virus type 2 (HSV-2), *Chlamydia trachomatis, Neisseria gonorrheae*, and *Trichomonas vaginalis* (10). Dendritic cells incubated with mucosal fluid from women with BV were found to up-regulate maturation and activation markers like HLA-DR, CD40, and CD83, and to have an increased T cell-stimulatory capacity indicating an impact on mucosal immunity (14). To determine if model pathogenic bacteria could similarly pereturb the complement-mediated avoidance of antiviral effects when DCs are exposed to bacteria, we added *Chlamydia* and opsonized HIV-1 either simultaneously mimicking a co-infection (HIV-C/Chlam-DCs) or by delayed addition of HIV-C (Chlam-DCs). *Chlamydia (C.) trachomatis* are gram-negative obligate intracellular bacteria and a primary agent causing non-gonococcal urethritis (15). During infection of cells within the vaginal mucosa, *C. trachomatis* initiates disruption of the mucosal-epithelial layer allowing better tissue entry of HIV-1 (10). Immunological alterations due to the presence of *C. trachomatis* may further support the transmission of HIV to susceptible cells or impact the antigen-presenting capacity of DCs (10).

Given that infection of iDCs is modulated by the opsonization pattern of HIV-1, which also had an impact on outcomes of both humoral and cellular antiviral immune responses (9, 16, 17) and given that HIV-1 particles are opsonized *in vivo* (18) and *in vitro* (4, 5), we analyzed whether the presence of *Chlamydia* modulates DC properties and function during co-infection with HIV-C.

## Materials and Methods

### Ethics Statement

This study was carried out in accordance with the recommendations of the Ethics Committee of the Medical University of Innsbruck. The protocol was approved by the Ethics Committee of the Medical University of Innsbruck. All subjects gave written informed consent in accordance with the Declaration of Helsinki.

### Generation of Human Monocyte-Derived iDCs and mDCs

Monocytes were isolated from whole blood of healthy donors by using CD14 BD IMAG Beads (Becton-Dickinson), according to the manufacturer's instructions. Differentiation into iDCs was done using IL-4 (200 U/ml) and GM-CSF (1,000 U/ml) and the iDC phenotype was routinely confirmed on day 5 by flow cytometry using CD11b, CD11c, DC-SIGN, HLA-DR, and CD83 (9, 16, 19). Representative histogram plots of these markers on iDCs are illustrated in Figure S1 (upper panel, red; isotype, blue). To generate LPS-DCs, day 5 iDCs were stimulated for 24 h with 100 ng/ml pure LPS-EB (Sigma) prior to HIV-1 infection.

Acute and chronic *Chlamydia* exposure was mimicked by stimulation of day 5 iDCs with infectious or heat-inactivated *Chlamydia* for either 24 h prior to (Chlam-DCs) or at the same time (HIV-C/Chlam-DCs or HIV/Chlam-DCs) as HIV-1 infection. For first experiments (DC maturation, binding, internalization) infectious as well as heat-inactivated bacteria were used. Since no differences were observed and since we intended to study PAMP-associated changes in DCs induced by *Chlamydia*, for all other analyses we used heat-inactivated bacteria. A representative histogram plot of CD83 expression on iDCs (red), DCs treated with heat-inactivated *Chlamydia* (dark green) or live *Chlamydia* (light green) is depicted in Figure S1, lower panel. Since isotype controls between the conditions did not differ, the iDC isotype control is shown (Figure S1, lower panel).

### Bacteria

*Chlamydia spp*. propagated in human epithelial HL cells and aliquots of purified bacteria were stored in sucrose phosphate glutamic acid at −80°C until use (20). For quantification of infection, coverslips overlaid with HL-cell-monolayers were fixed in methanol and stained with FITC-conjugated anti-Chlamydia LPS monoclonal antibody (OXOID (Ely) Ltd., Ely, UK). Chlamydial inclusion bodies within cells were counted by fluorescence microscopy at a magnification of x100 with a Scope A1 microscope (Zeiss). For experiments using infectious, purified *Chlamydia* cells were infected at a multiplicity of infection/MOI of 10 as described earlier (20). An aliquot of tested, purified *Chlamydia spp*. was used for heat-inactivation at 70°C for 20 min.

### Plasmids

The infectious R5-tropic HIV-1 proviral clone R9Bal was used for maturation, binding/internalization and DC infection studies. For HIV-1 fusion assays the R9Bal and vpr/β-lam expression constructs were used to generate chimeric R9Bal/β-lam pro-viral clones. Confocal microscopic analyses and HC/HT imaging analyses were performed by using chimeric R9Bal/mCherry virus preparations originating from R9Bal and vpr/m-Cherry expression plasmids (21).

### Virus Production

HIV-1 proviral clones were produced by transfecting HEK293T cells. R9Bal/β-lam and R9Bal/mCherry virus stocks were prepared by co-transfection of HEK293T cells with the pro-viral R9Bal DNA and the vpr-β-lam or vpr-mCherry expression constructs (9). Freshly produced virus was obtained via ultracentrifugation (70,000 × g/90 min/4°C). Concentration of the ultracentrifuged virus was measured by p24 ELISA (22) and viral infectivity was confirmed by the determination of the TCID_50_ using PHA/IL-2-stimulated PBLs. To monitor productive infection of DCs or DC/T cell co-cultures, p24 ELISA was used.

### Opsonization of Viral Stocks

Viruses were opsonized by incubation with normal human serum (NHS) as a complement (C) source in a 1:10 dilution for 1 h at 37°C (HIV-C). As negative control, the viruses were incubated under the same conditions in medium, which reflects non-opsonized HIV-1 (HIV). After opsonization, viruses were thoroughly washed, pelleted by ultracentrifugation (25,000 × g/90 min/4°C) and re-suspended in RPMI medium. The opsonization pattern was determined by virus capture assay (VCA) as described (7). 96-well high-binding plates were coated using anti-human C3c, C3d, or IgG antibodies. Mouse IgG antibody was used as a control for background binding. Plates were then incubated overnight at 4°C with the differentially opsonized virus preparations (10 ng p24/well). After extensive washing, virus was lysed and p24 ELISA was performed.

### Capture of HIV-1

Differentially matured DCs (1 × 10^5^ cells/well/100 μl) were exposed to 25 ng p24/ml of R5 tropic non-opsonized (R9Bal) or complement-opsonized (R9Bal-C) HIV-1. After 6 h incubation at either 4°C for binding or 37°C for internalization, cells were washed 4 times to remove unbound virus. Cell pellets were lysed with 2% Igepal and viral amount was assessed by p24 ELISA.

### Viral Fusion Assay

DCs were plated into 96-well plates (1 × 10^5^ cells/well/100 μl) and infected with 250 ng p24/ml non-opsonized or opsonized R9Bal/β-lam. After 5 h incubation cells were washed and loaded for 1 h with CCF2-AM substrate solution according to the manufacturer's instructions (LiveBLAzer™ FRET-B/G Loading Kit with CCF2-AM, LifeTechnologies). Cells were washed again and developed for 16 h in CO_2_-independent medium (Gibco) containing 10% FCS and 2.5 mM probenicid. Cleavage of CCF2 was analyzed by flow cytometry after fixation of DCs in 4% paraformaldehyde.

### Microscopy

To visualize intracellular HIV-1 localization by confocal microscopy, iDCs, HIV-C or HIV/Chlam, Chlam- and LPS-DCs were plated onto Poly-L-lysine (Sigma)-coated coverslips and exposed to R9Bal/mCherry or –GFP (350 ng p24/ml) for 24 h. For HC/HT screening analyses, various matured DCs (50,000/well) were seeded in CellCarrier Ultra plates (Perkin Elmer) and infected over night with fluorescently labeled HIV-C (350 ng p24/ml). DCs were fixed with 4% paraformaldehyde, labeled using Hoechst 33342 (Cell Signaling Technologies), permeabilized (Permeabilization Wash Buffer, BioLegend), and stained with HLA-DR (BioLegend). Following staining, cells were washed and mounted (confocal microscopy) or re-suspended in D-PBS (HC/HT Screening). Confocal microscopy was performed on a Leica SP5 (Leica Microsystems) using a glycerol objective. Images were analyzed using LAS AF Lite (Leica Microsystems) and Fiji (ImageJ). For 3-D-rendered stacks, Imaris (Bitplane) was used. HC/HT analyses were performed using an Operetta CLS™ (Perkin Elmer) and co-localization of mCherry/HLA-DR or GFP/Siglec-1 automatically quantified using the Harmony™ Software and RMS Spot Analyses (Perkin Elmer). For these automated analyses, first fluorescence intensities were measured, since if HIV particles are in the cytoplasm, the fluorescence intensities are significantly lower compared to packed virus in endosomes. Lower intensities can then be excluded from the automatic screening process. Then co-localization of virus particles with HLA-DR, which is in endosomal compartments only, was measured.

### DC Infection

Day 6 iDCs, HIV-C or HIV/Chlam-DCs, Chlam-DCs and LPS-DCs (1 × 10^5^cells/well/100 μl) were infected in triplicate with 25 ng p24/ml of R9Bal or R9Bal-C. After 24 h incubation, DCs were thoroughly washed and cultured at 37°C and 5 % CO_2_ for 15 days. For co-infection experiments, autologous CD4^+^ T cells were added to washed DCs the day after HIV-infection. After several days post-infection, supernatants were taken and diluted 1:10 with 2% Igepal to lyse the virus. Productive infection was determined by measuring p24 concentrations in the supernatant.

### Interferon-γ Elispot

SL9 clone 2, a HIV-specific CD8^+^ CTL clone, was derived from an HIV-infected patient and recognizes the well-characterized immune-dominant epitope of Gag p17 SLYNTVATL (SL9) presented by HLA-A^*^02:01 (23, 24). The human immune response to the HLA-A^*^02:01-restricted Gag_77−85_ SLYNTVATL epitope is the most studied—SL9 is a highly immunogenic, help-independent HIV-1 epitope and a strong negative association was demonstrated between SL9-CTL levels and viral load (25). DCs were co-cultured overnight with SL9-CTLs (2,500–10,000 clones/well). As positive controls, DCs were incubated with 1 μg/ml of cognate peptide before washing and addition of the HIV-specific CTL clones overnight. IFN-γ production was monitored in an Elispot assay as described (24). All antibodies (Abs) used for the IFN-γ Elispot were purchased from Mabtech.

### Multicolor FACS Analyses

Differentiation and maturation of DCs exposed to *Chlamydia* or LPS and HIV-1 were analyzed by using anti-human CD11c-AlexaFluor488, HLA-DR-PerCP/Cy5.5, DC-SIGN-PE (Biolegend), CD86-BV421, CD83-APC, CD169-PE (BD Biosciences) on a FACS Verse flow cytometer (BD Biosciences). Cell surface expression of receptors for HIV and HIV-C binding was determined by flow cytometry as described using anti-human CD11b-APC, CD4 PerCP/Cy5.5 and DC-SIGN-PE (BioLegend). Data was analyzed using FACS DIVA software (BD Biosciences) and R.

### Statistical Analysis

Data were analyzed using GraphPad Prism software (GraphPad Software Inc.). Statistical analyses were performed using two-way ANOVA with Dunnett's posttest for multiple comparisons.

## Results

### Reduced Maturation of DCs During Chlamydial Co-infection Compared to Sequential Infection

**Figure 1:**
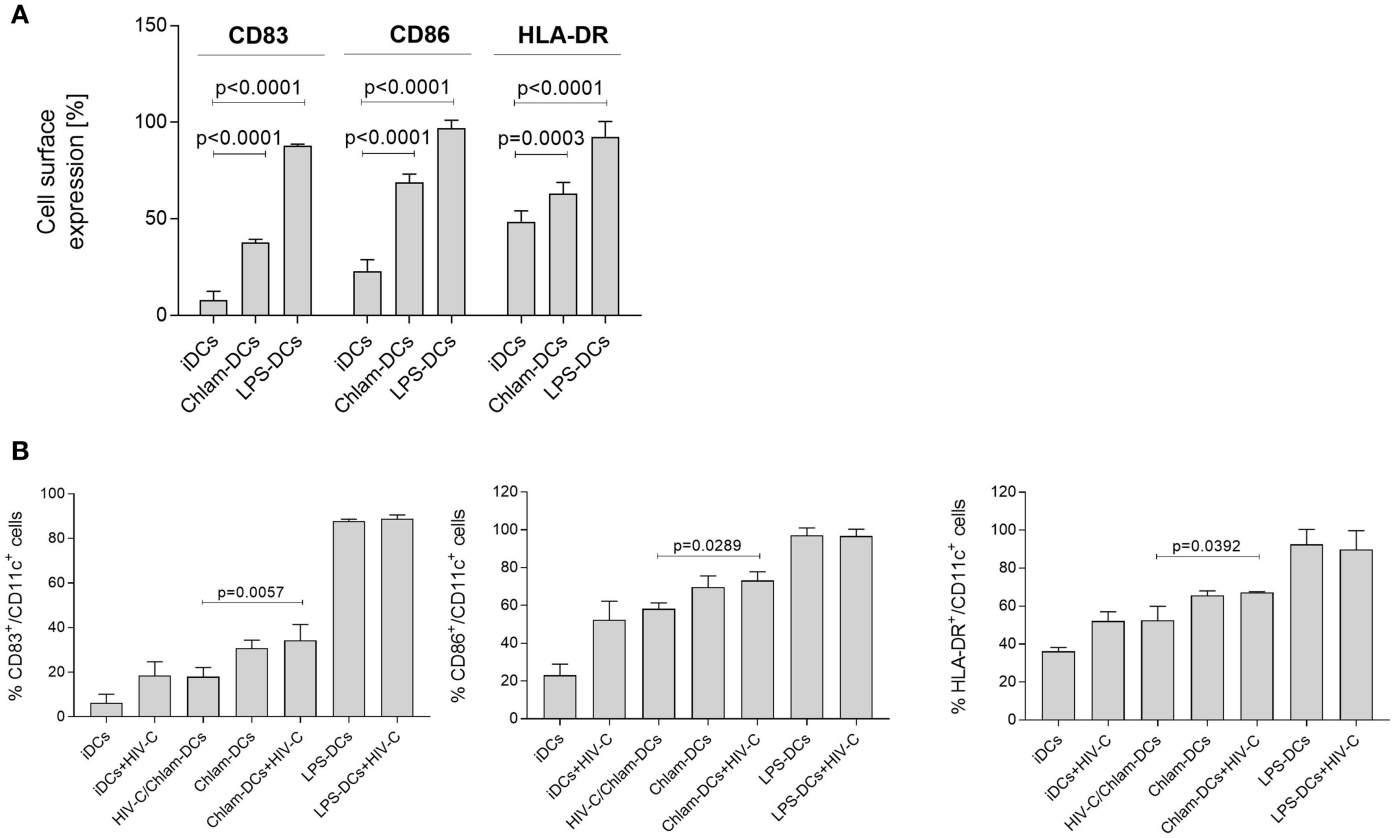
*Chlamydia* induces maturation of DCs *in vitro*. **(A)** Representative flow cytometric analysis of CD83, CD86, and HLA-DR expression on CD11c^+^ DCs upon stimulation with *Chlamydia* or LPS for 24 h. Percentages+/-SD of double positive (CD83^+^/CD11c^+^, CD86^+^/CD11c^+^, HLA-DR^+^/CD11c^+^) DCs are indicated for 6 independent donors. **(B)** CD83 (left), CD86 (middle) and HLA-DR (right panel) expression levels are not changed in co- or sequential infected cells. Levels of HIV-C/Chlam-DCs and HIV-C-DCs are similar, while delayed addition of HIV-C to Chlam-DCs represents the maturation and activation status of Chlam-DCs. LPS-DCs were used as positive controls. To simplify the graph, statistics are only depicted for differences between the co- and sequential infection model. Percentages+/-SD of double positive (CD83^+^/CD11c^+^, CD86^+^/CD11c^+^, HLA-DR^+^/CD11c^+^) DCs are indicated for 3 donors. Statistical analysis was performed using 2way ANOVA and Dunnett's multiple comparisons test.

We initially evaluated whether exposure to C*hlamydia* induced maturation of DCs similarily to the positive control LPS. Therefore, we analyzed cell surface expression of the specific markers CD83, CD86 and HLA-DR after the different treatments. We found that long-term exposure (24 h) of iDCs to *Chlamydia* induced significant up-regulation of CD83, CD86, and HLA-DR compared to untreated iDCs (Figure 1A). However, expression levels of CD83, CD86, and HLA-DR on Chlam-DCs were lower compared to LPS-stimulated DCs (LPS-DCs) in all donors tested (Figure 1A, *n* = 6). Independent of DC stimulation, the expression levels of DC-SIGN and the complement receptors 3 and 4 (CR3, CD11b/CD18; CR4, CD11c/CD18) were only moderately changed and CD4 expression was slightly reduced under all maturation conditions as also shown by Chen et al. (26) (not shown). Exposure of such various matured DCs (Chlam-DCs, LPS-DCs) to HIV-C did not change the expression of up-regulated markers CD83 (Figure 1B, left panel), CD86 (Figure 1B, middle panel), and HLA-DR (Figure 1B, right panel). In contrast, a reduced maturation of DCs was observed upon co-infection with HIV-C and *Chlamydia* (HIV-C/Chlam-DCs) and this maturation was comparable to that when iDCs were exposed to HIV-C only (Figure 1B, CD83—left panel, CD86—middle panel, HLA-DR—right panel). Expression of all maturation and activation markers was significantly higher on HIV-C- and HIV-C/Chlam-DCs compared to iDCs. We demonstrated that stimulation of DCs with *Chlamydia* caused a lower DC maturation compared to LPS and this maturation was not increased due to additional HIV-C exposure.

### Binding of HIV-C Depends on the DC Maturation Status

**Figure 2:**
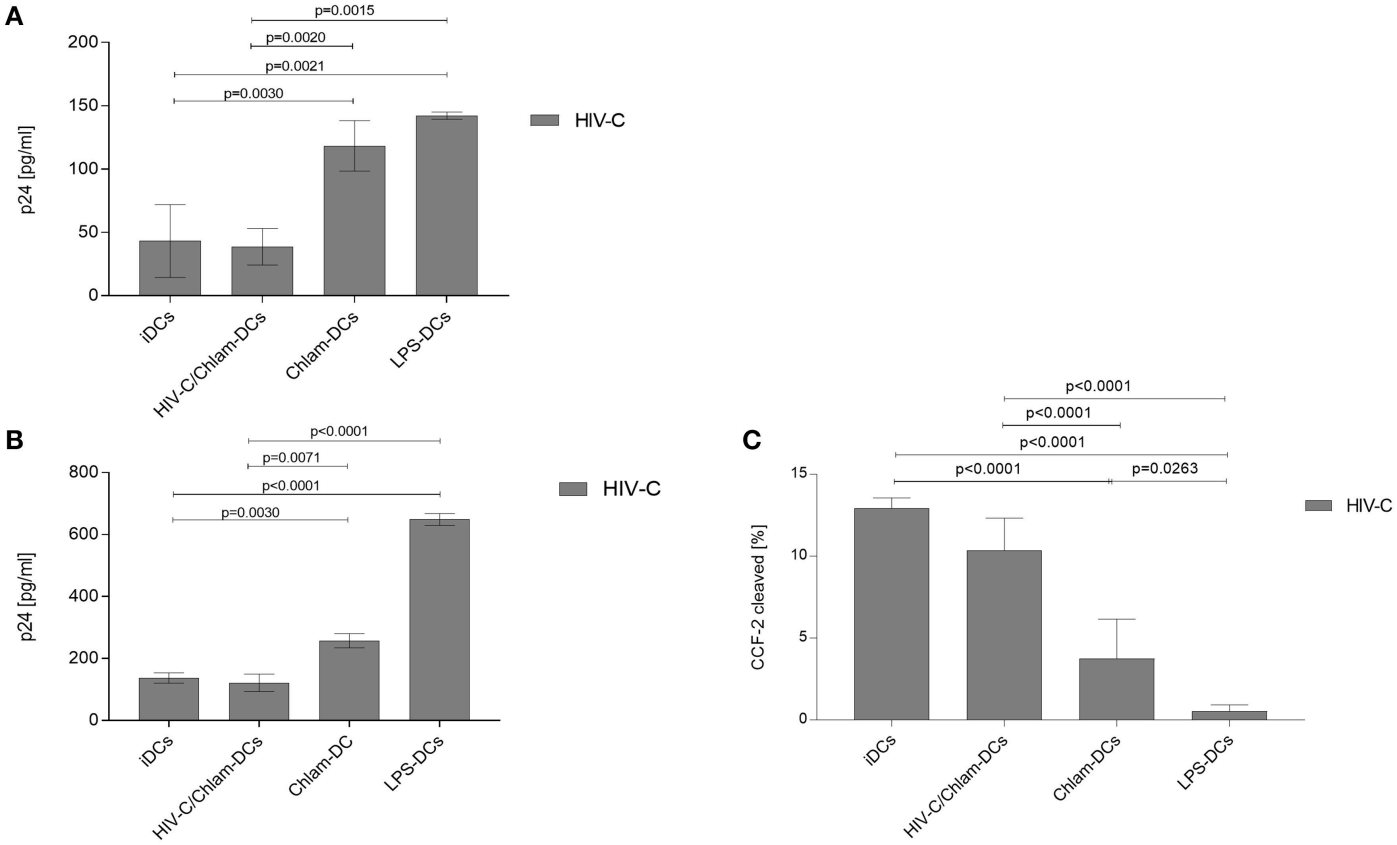
Chlam- and LPS-DCs efficiently capture HIV-C. Binding at 4°C **(A)** and internalization at 37°C **(B)** were performed in triplicates using 25 ng/ml of R5-tropic opsonized HIV-1. Bar graphs show means ± SD from three independent experiments. p24 levels within the cell lysates were determined by ELISA. Prior to cell lysate preparation, cells were thoroughly washed to remove unbound virus. Statistical analysis shows 2-way ANOVA with Tukey's multiple comparisons test. **(C)** Fusion assays were performed after addition of HIV-C bearing the chimeric protein β-lactamase-Vpr to iDCs, Chlam-DCs, and LPS-DCs or after simultaneous HIV-C-VprBlam/Chlamydia stimulation of iDCs (HIV-C/Chlam-DCs). The amount of fused virus was determined by flow cytometric analyses of cleaved CCF2 in the cytoplasm. Percentages of cleaved CCF2-positive cells from three independent donors are depicted.

Since expression of activation markers was shown to be different on iDCs, Chlam- and LPS-DCs, we assessed whether this might lead to differential binding of HIV-C to DCs. To characterize binding of HIV-C co-cultures of various matured DCs and HIV-C were incubated for 6 h at 4°C (8). At 4°C, DCs just bind but do not internalize viral pathogens (8). HIV-C (25 ng p24/ml) was added to iDCs, Chlam- and LPS-DCs for 6 h at 4°C. Using the co-infection model, DCs were incubated with simultaneously added HIV-C and *Chlamydia* under above mentioned conditions. Cell-bound virus was determined after thorough washing and lysing of DCs by quantification of p24 protein. Similar amounts of HIV-C were attached to iDCs and HIV-C/Chlam-DCs, while Chlam- and LPS-DCs depicted a significantly increased binding of HIV-C (Figure 2A). A similar binding pattern was analyzed for non-opsonized HIV-1 (HIV, Figure S2A). Therefore, binding to DCs was independent of the opsonization pattern, but was modulated by DC maturation status.

### DC Maturation Affects HIV-C Internalization

To also see if internalization of HIV-C in iDCs, HIV-C/Chlam-, Chlam-, and LPS-DCs differs, we incubated differentially stimulated cells for 6 h at 37°C. Virus was added as described above and bound/internalized HIV-C was determined by p24 ELISA after washing and lysing the cells. These analyses revealed that LPS-DCs show a ~5-fold higher internalization compared to iDCs and HIV-C/Chlam-DCs (Figure 2B). Internalization of HIV-C into LPS-DCs was significantly higher compared to its non-opsonized counterpart (Figure S2B, *p* = 0.005). Though the internalization of HIV-C in Chlam-DCs was lower compared to LPS-DCs, a significantly higher internalization of HIV-C compared to both iDCs (*p* = 0.0030) and HIV-C/Chlam-DCs (*p* = 0.0071) was identified (Figure 2B). The increase in HIV-1 internalization upon DC maturation was observed independent on whether the virus was opsonized (Figure 2B) or not (Figure S2B).

### DC Maturation Affects HIV-C Fusion

To further evaluate the impact of iDC maturation by the different treatments on the interaction of cell and virions, we analyzed virion fusion using Vpr-β-lactamase (Vpr-blam)-containing HIV-C (Figure 2C) or HIV (Figure S2C). We found that fusion was not inhibited in HIV-C/Chlam-DCs relative to HIV-C-exposed iDC controls (Figure 2C). In contrast fusion was significantly decreased in Chlam-DCs and LPS-DCs (Figure 2C). It is notable that fusion was completely inhibited in the LPS-DCs independent of the opsonization pattern of the virus (Figure 2C and Figure S2C). iDCs and co-infection of DCs with *Chlamydia* were associated with the highest fusion with HIV-1, while sequential infection with *Chlamydia* displayed significantly lower fusion levels with a complete inhibition of fusion in LPS-DCs.

### Siglec-1 Does Not Play a Role With Respect to HIV-C Capture

Since Siglec-1 (CD169) was described—at least *in vitro*- to exert a prominent role with respect to capture and transfer of HIV-1 in LPS-stimulated mDCs (27–29), we analyzed co-localization of this molecule with GFP-tagged HIV or HIV-C in differently stimulated DCs. For this, we performed high content screening of differentially stimulated and infected DCs and analyzed the co-localization of GFP-tagged virus with PE-labeled Siglec-1. We automatically analyzed two fields á 100 cells for their co-localization of HIV-1 and Siglec-1 using the Harmony^™^ software (Perkin-Elmer) and mean values of spots co-localizing within 100 cells are depicted in Figure S3. These analyses revealed no significant differences but only slightly higher Siglec-1/HIV-C co-localization in Chlam-DCs compared to iDCs or HIV/Chlam-DCs and compared to background fluorescence of non-infected cells, which served as negative controls (Figure S3). As positive controls, mature DCs (Chlam-DCs or LPS-DCs) infected with non-opsonized HIV-1 (HIV) were used, which displayed significantly higher co-localization compared to HIV-C-infected DCs. the results suggest that a modulation of the interaction of HIV-1 and Siglec-1 is not playing a major role in viral capture.

### HIV-C Localizes to HLA-DR-Containing Compartments in Chlam- and LPS-DCs

**Figure 3:**
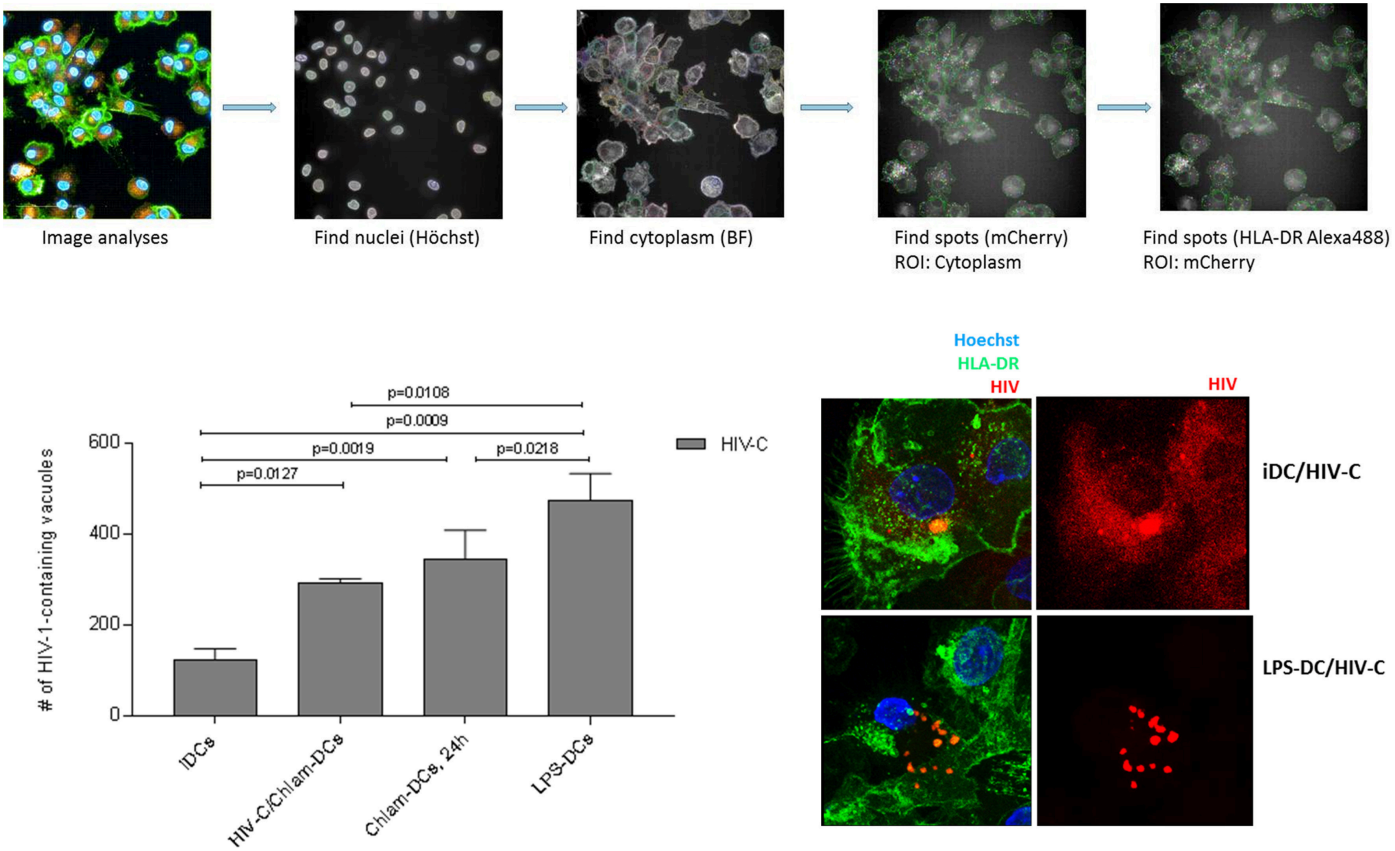
HIV-C/Chlam-, Chlam-, and LPS-DCs show significantly higher levels of vacuolar HIV-C. Intracellular localization of HIV-C in LPS-DCs was investigated by HC/HT screening and confocal microscopic analyses. The path of image analyses is illustrated in the upper panel. Briefly, nuclei (Hoechst) and cytoplasm (Brightfield) of differentially matured and HIV-C-infected DCs were identified using the Harmony 4.6 software (Perkin Elmer). Within at least 50 to 100 scanned cells, mCherry-labeled HIV-spots co-localizing with Alexa488-labeled HLA-DR were identified. Quantitative analyses of HIV-C-containing HLA-DR-positive vacuoles are depicted in the lower left panel. Co-localization of HIV-C and HLA-DR in LPS-DCs was further confirmed using confocal microscopic analyses. iDCs show, besides some compartmentalized HIV, randomly distributed cytoplasmic HIV-C (red) while LPS-DCs display solely concentrated HIV-C within cellular compartments.

To gain additional insights into potential differences in the interaction of HIV-1 with iDCs matured by the different treatments, we evaluated the intracellular localization of HIV-C in iDCs, HIV/Chlam-, Chlam-, and LPS-DCs. To this end, we infected the respective different DC populations using fluorescently labeled HIV-C and analyzed viral particle distribution of internalized HIV-C by high-content/high-throughput (HC/HT) image analyses and confocal microscopy (Figure 3, Figure S4). For these analyses, cells were additionally labeled using a nuclear stain (Figure 3, Figure S4, Hoechst, blue) and HLA-DR as marker for endosomal compartments including virus containing compartments (VCCs) (Figure 3, Figure S4, green). The analyses revealed significantly lower levels of HIV-C-containing HLA-DR-containing compartments in iDCs compared to HIV-C/Chlam-, Chlam-, and LPS-DCs (Figure 3, left, histogram plot). Significantly higher HLA-DR-containing compartment levels were detected in LPS-DCs compared to both, HIV-C/Chlam- and Chlam-DCs (Figure 3, left, histogram plot). The accumulation of the virus in HLA-DR-containing compartments in LPS-DCs was confirmed using confocal microscopic analyses (Figure 3, right)—these revealed vacuolar and cytoplasmic distribution of HIV-C in iDCs (Figure 3, right, upper panel, Figure S4, left), while only HLA-DR-containing compartments, but no cytoplasmic HIV-1, were detected in LPS-DCs (Figure 3, right, lower panel, Figure S4, right). Figure 3 shows volume projections of maximal pixel intensity of all layers analyzed and Figure S4 the respective 3D-rendered z-stacks. Fusion and image analyses by HC/HT screening and confocal microscopy revealed a high cytoplasmic distribution of HIV-C in iDCs and under conditions of co-infection. The cytoplasmic distribution was reduced in sequentially infected DCs and completely abrogated in LPS-DCs. In contrast HIV-1-containing vacuoles were mainly detected in LPS-exposed DCs, to lower levels in *Chlamydia*-exposed DCs and only marginally in iDCs.

### DC Infection Is Enhanced by *Chlamydia* Co- and Sequential Infection

**Figure 4:**
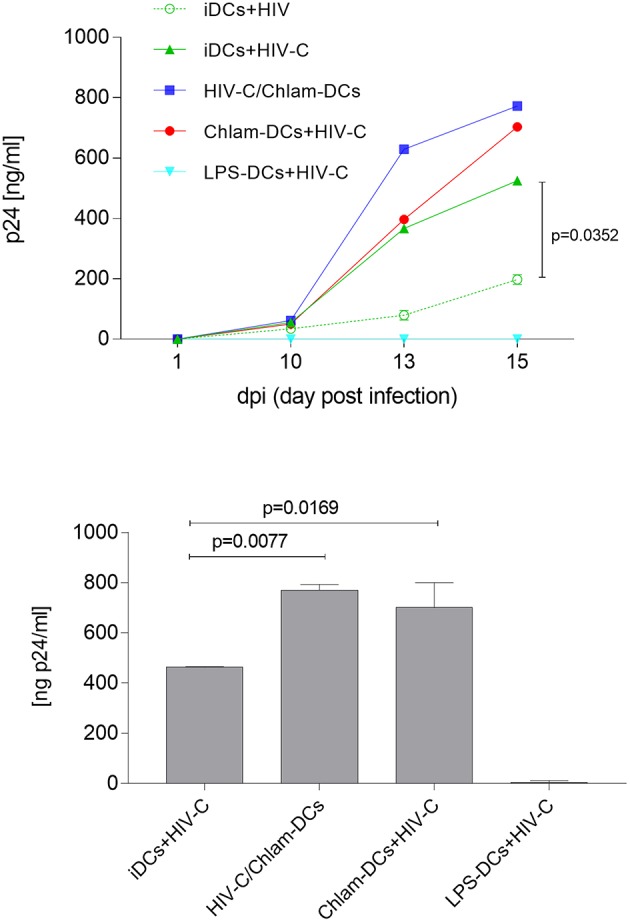
Enhanced productive infection of Chlam-DCs mediated by HIV-C. DCs were infected with 25 ng p24/ml of HIV-C. The graph depicts an infection course of DCs—p24 levels (means ± SD) within the supernatant were analyzed on day 1-10-13 and 15 post-infection. Highest productive infection was measured in supernatants from HIV-C-infected HIV-C/Chlam- (blue) and Chlam-DCs (red) followed by HIV-C-exposed iDCs (green, solid line). Non-opsonized HIV caused the already described low-productive infection in DCs (green, dashed line) (9). No infection was detected in LPS-DCs independent of the opsonization pattern of the virus (turquoise). The upper panel shows the kinetics of one out of three representative assays including technical triplicates, while in the lower panel all three donors were combined and day 15 post infection is depicted.

We recently demonstrated that HIV-C overcomes restriction in iDCs resulting in significantly higher productive DC infection, improved antigen-presentation as well as humoral antiviral immune responses (30). We analyzed productive DC infection using HIV-C in co- (HIV+Chlam-) and sequential infection (Chlam-) DC models as well as LPS-DCs. iDCs were used as controls—again we found that non-opsonized HIV caused a significantly lower productive infection of iDCs (Figure 4, upper panel, dotted green line, HIV, vs. green line, HIV-C and Figure S5) compared to HIV-C despite similar binding and internalization (Figure 4, upper panel, and Figure S1). However, neither HIV (not shown) nor HIV-C (Figure 4, upper and lower panels) caused any productive infection in LPS-DCs. Within the co-infection model, both HIV-C (Figure 4, upper and lower panels) and HIV (Figure S5) exerted an enhanced productive DC infection compared to iDCs (Figure S5)—nevertheless, complement opsonization of HIV-1 still promoted a significantly increased DC infection compared to its non-opsonized counterpart (Figure S5). Although Chlam-DCs—representing the sequential infection—showed a high maturation and low viral fusion, they were infected to high levels with both HIV-C (Figure 4) and HIV (Figure S5). These data suggest that HIV-C facilitated productive infection in DCs during chlamydial co- and sequential infection as well as a different maturation status between LPS- and *Chlamydia*-matured DCs.

### HIV-C*/Chlamydia* Co- but not Sequential Infection of DCs Is Associated With Reduced HIV Transfer

**Figure 5:**
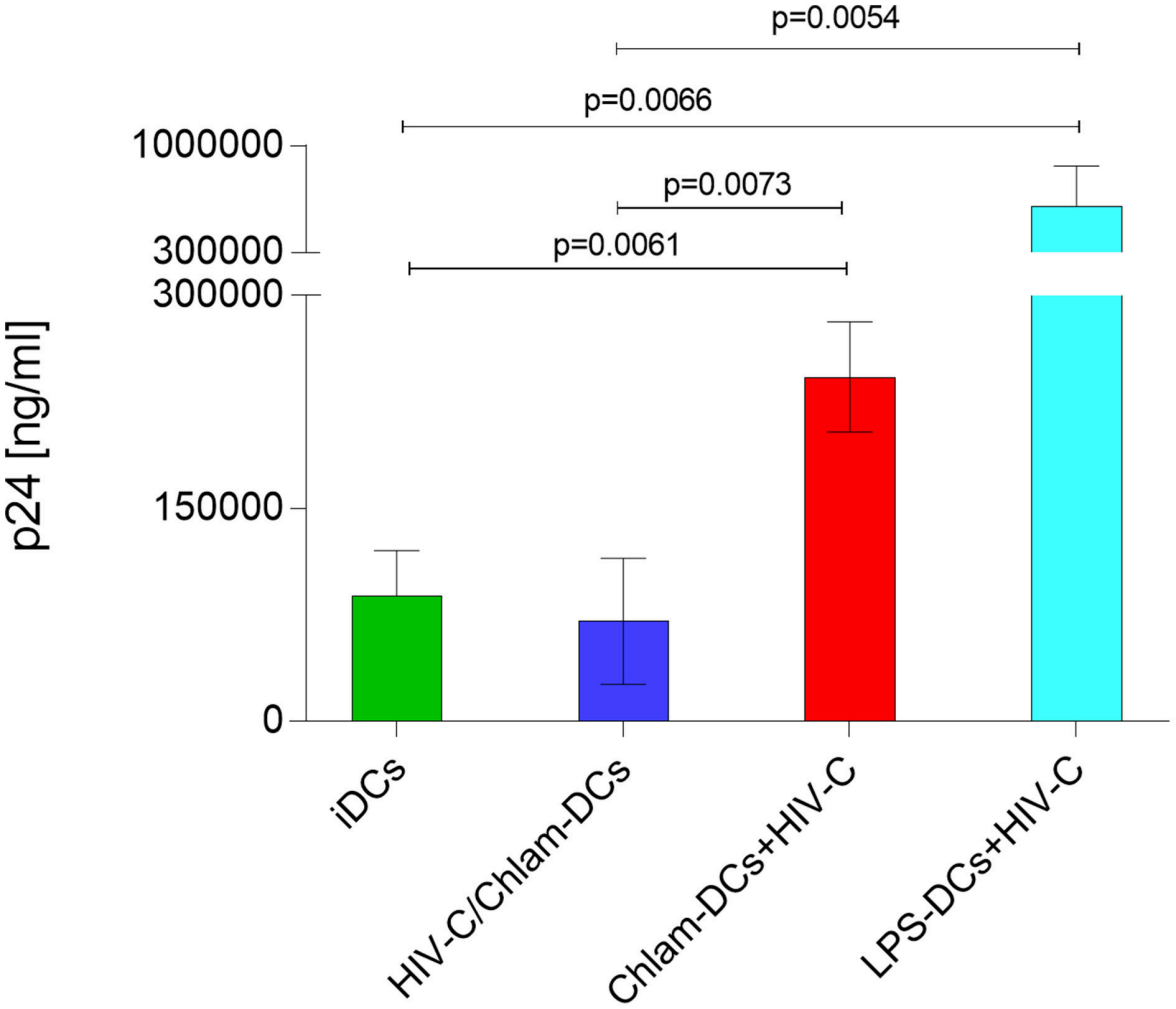
HIV-C is efficiently transferred from Chlam- and LPS-DCs. In co-culture experiments differentially stimulated DCs (iDCs, green; HIV/Chlam-DCs, blue; Chlam-DCs, red; LPS-DCs, turquoise) were infected using HIV-C (25 ng p24/ml), thoroughly washed and autologous CD4^+^ T cells were added. Significantly higher infectivity was measured in Chlam-DC- and LPS-DC co-cultures compared to iDC- and HIV-C/Chlam-DC-CD4^+^ T cell co-cultures. p24 ELISAs of differently stimulated DC/T cell co-cultures performed in triplicates from two donors exposed to HIV-C are summarized and statistical analyses were performed using two-way ANOVA with Dunnett's posttest for multiple comparisons.

Lastly, we evaluated HIV-1 trans-infection from differently matured DCs to autologous, stimulated CD4^+^ T cells as revealed by a co-culture with T cells. In these studies, we found that simultaneous stimulation of DCs with HIV-C and *Chlamydia* resulted in similar infection rates to CD4^+^ T cells as HIV-C-exposed iDCs. Compared to Chlam- and LPS-DCs, these conditions illustrated significantly reduced trans-infection in co-culture experiments (Figure 5). As demonstrated previously for non-opsonized HIV (27, 31), LPS-matured DCs, too, transmitted significantly more virus when complement-opsonized compared to iDCs, HIV/Chlam- and Chlam-DCs (Figure 5). Therefore, levels of transmitted HIV-C in *Chlamydia*-matured DCs differ in co- and sequential infection models and transfer does not correlate with Siglec-1 co-localization in the HIV-C model (Figure S3).

### *Chlamydia* Co-infection Promotes Significant Activation of HIV-Specific CTLs, While Reversing the Situation During Sequential Infection

**Figure 6:**
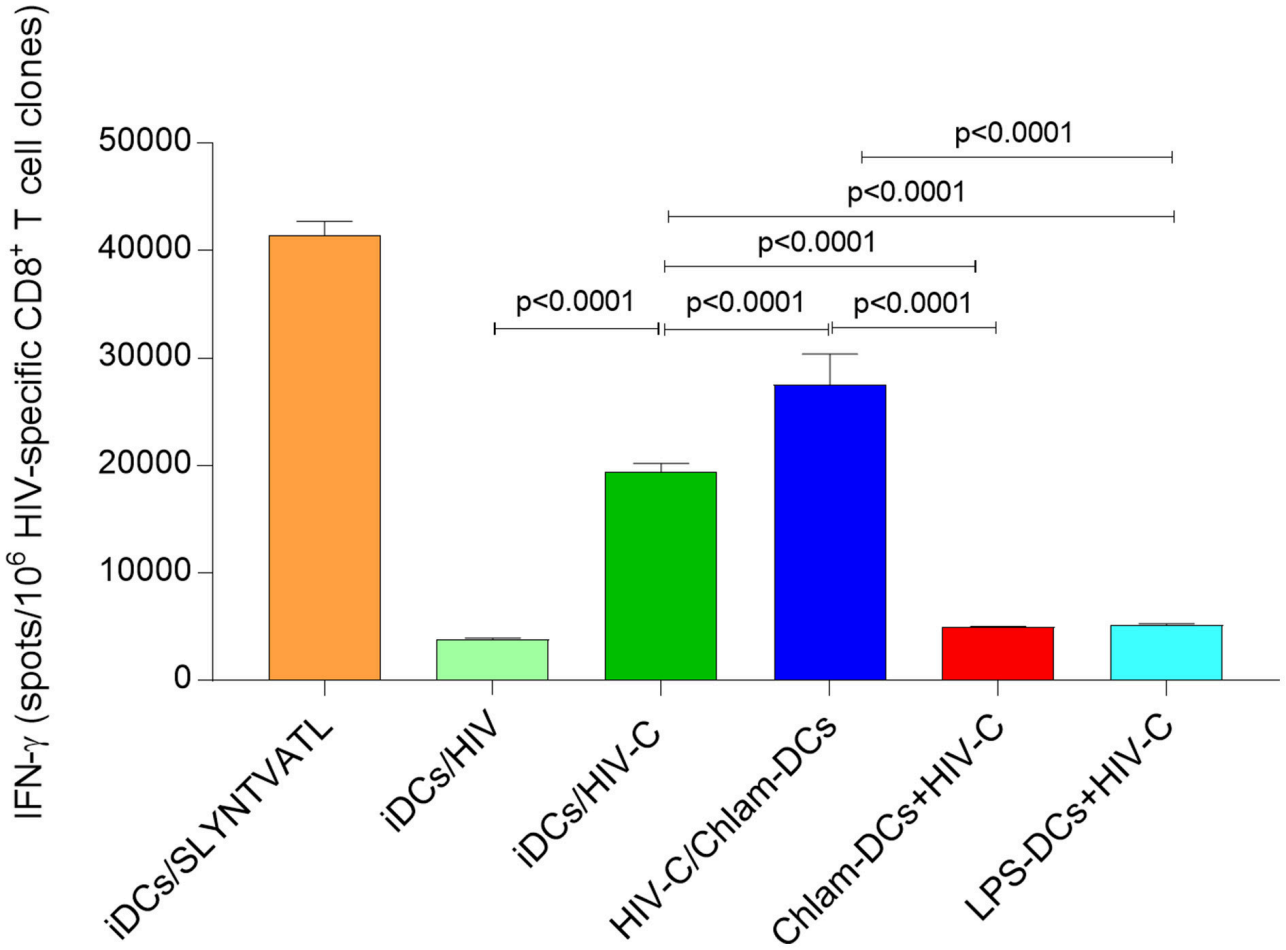
Enhanced stimulation of HIV-specific T cell clones at simultaneous addition. IFNγ induction in CD8^+^ T cell clones by HIV-C-exposed iDCs and HIV-C/Chlam-DCs was significantly stronger than that of non-opsonized HIV-loaded DCs (HIV-DCs; *p* < 0.0001 for CD8^+^ T cell clones), or Chlam- and LPS-DCs exposed to HIV-C (*p* < 0.0001 for all). As positive controls specific peptide-loaded DCs for CD8^+^ T cell clones were used (iDCs/SLYNTVATL). IFNγ Elispots of CD8^+^ T cell clones were repeated using HLA-matched and differently stimulated DCs from three donors exposed to HIV-C, or HIV. Statistical analyses were performed using two-way ANOVA with Dunnett's posttest for multiple comparisons.

Bypassing of restriction mechanisms in iDCs and enhanced productive infection using HIV-C rendered the cells capable to activate highly specific anti-HIV-cellular and humoral immune responses (9, 17). To determine the potential impact of *Chlamydia* on cellular HIV responses, we evaluated the ability of differently matured DCs (iDCs, HIV-C or HIV/Chlam-, Chlam-DCs, and LPS-DCs) exposed to HIV-C (Figure 6) or HIV (Figure S6) to stimulate HLA-matched HIV-specific CTLs. While in the co-infection model, when *Chlamydia* and HIV-C were added simultaneously, we detected a significantly higher CTL stimulatory capacity compared to HIV-C-exposed iDCs (Figure 6). Within the sequential infection model (Chlam-DCs, LPS-DCs) a significantly abrogated potential to stimulate HIV-specific CD8^+^ T cells was observed (Figure 6). SLYNTVATL-exposed DCs were used as positive controls (Figure 6). The CTL-stimulatory power of DCs was also drastically augmented using co-infection of the cells with bacteria and non-opsonized HIV-1 (Figure S6). As already observed during our earlier work, HIV-loaded iDCs exerted a very weak CTL-stimulatory capacity (Figure S6) (9, 18). These observations illustrate that co-infection of DCs with *Chlamydia* and HIV-C or HIV is associated with induction of HIV-specific CTL responses, while sequential infection results in increased hazard with respect to the weak CTL-stimulatory capacity of DCs.

## Discussion

The studies presented here reveal that infection of the host with *Chlamydia* and HIV-1 have both potential positive and negative impact on HIV infection. Simultaneous infection of DCs with *Chlamydia* and HIV might be beneficial for the host as this triggers a higher HIV-specific CTL activation and lower transfer of HIV to autologous CD4^+^ T cells. In contrast, sequential infection of DCs with *Chlamydia* and HIV, which might be a common situation in the host, results in detrimental outcomes as it is associated with higher productive DC infection and viral transmission to susceptible CD4^+^ T cells as well as poorer stimulation of HIV-1-specific CD8^+^ T cell clones.

Upon simultaneous stimulation of DCs with *Chlamydia* and either complement-opsonized HIV-1 or untreated control HIV, a significantly improved CTL response was observed. This is in contrast to the requirement for complement-opsonization we previously reported in the absence of *Chlamydia* exposure to act as an endogenous adjuvant for DC-mediated CTL activation of iDC (16). We also find that HIV-exposed DCs co-infected simultaneously with *Chlamydia* exerted a superior CTL-stimulatory capacity of HIV-specific CD8^+^ T cell clones compared to their HIV-iDC counterparts (9, 16, 18). As shown recently in a murine vaginal co-infection model (32), chlamydial pre-infection protected the mice from subsequent Herpes Simplex Virus (HSV)-2 challenges. This *Chlamydia*-mediated protection was transient and only detectable in mice pre-challenged with *Chlamydia* before, simultaneously with, or shortly after infection with HSV-2 (32). These findings are in accordance with our data, where co-infection of DCs with *Chlamydia* and HIV or HIV-C resulted in significantly higher CTL activation via DCs. In contrast, DC sequential infection for 3 h or 24 h with *Chlamydia* followed by HIV-C infection had detrimental outcomes (Figures 4, 5). Under these conditions, sequentially infected DCs only had a poor capacity to stimulate HIV-specific CTLs and allowed significantly higher productive HIV infection (*cis* infection). In contrast, no *cis*-infection was analyzed at all in DCs challenged for 3 or 24 h with LPS prior infection with HIV-C. The impact of pre-existing STIs on HIV immune responses was studied by Sheung et al. (33) in high risk Kenyan female sex workers. They found that mucosal *Neisseria gonorrhoeae* co-infection during HIV-1 acquisition was associated with substantially enhanced HIV-specific CD8^+^ T cell responses (33). The enhanced CTL response was not seen in women with *Chlamydia* co-infection, which correlates well with our findings within the sequential infection model of DCs with *Chlamydia* and HIV-1, which exerted a weak HIV-specific CTL activation. However, to study the impact of simultaneous STI on HIV immune responses is logistically impossible in the human host.

LPS-DCs had the highest binding and internalization of HIV-C followed by Chlam-DCs, while iDCs and HIV-C/Chlam-DCs showed similar HIV-C up-take levels. As described earlier, maturation of DCs—as seen in LPS- or Chlam-DCs—enhances their virus capture and *trans* infection capacity while reducing viral fusion events (34). HIV-C/Chlam-DCs are not as mature as Chlam-DCs, when binding and internalization were measured. Therefore, HIV-C/Chlam-DCs more act like iDCs, which show less binding and internalization, but enhanced fusion. Consistent with this interpretation, the highest levels of fusion were measured in iDCs and HIV-C/Chlam-DCs, while Chlam- and LPS-DCs demonstrated considerably reduced fusion levels (34). Consistent with our fusion data, the accumulation of HIV-C in HLA-DR-containing compartments was highest in LPS-DCs and also Chlam-DCs showed significantly higher HIV-C-containing compartments compared to iDCs. In macrophages, virus containing compartments (VCCs) were described to resemble late endosomes or multi-vesicular body (MVB) compartments and to show enrichment of CD9, CD53, CD81, CD82, and MHC class II (35, 36). We previously illustrated co-localization of HIV-C with these markers (7). VCCs are non-acidic and often express surface-connected tubular conduits to the plasma membrane (35, 37, 38). VCC formation was demonstrated to greatly facilitate *trans*-infection of HIV-1 from macrophages to autologous CD4^+^ T cells (39). Accumulation of viral particles within intracellular DC compartments was illustrated to share multiple features with macrophage VCCs (30, 40, 41). Concentration of non-opsonized HIV-1 particles in large sac-like and tetraspanin-rich/MHC II compartments within LPS-mDCs was shown by various imaging studies (27, 42, 43). We also show a similar distribution of HIV-C in MHC II (HLA-DR-) compartments particularly in Chlam- and LPS-matured DCs. Transfer of such trapped viral particles, which were non-opsonized, from mDCs to CD4^+^ T cells was highly effective (44–46). Localization of internalized virus differs greatly in endocytically active iDCs compared to mDCs—mDCs storing intact HIV particles within large vesicles correlate with increased *trans*-infection abilities (34). We here demonstrate (47), that similar to non-opsonized HIV-1, mature DCs (i.e., LPS-DCs and Chlam-DCs) retained HIV-C particles in an infectious form and efficiently transmitted the virus particles to target CD4^+^ T cells through *trans* infection. Despite co-infection with *Chlamydia*, DCs displayed significantly higher amounts of trapped virus particles compared to iDCs loaded with HIV-C. Such co-infected DCs exerted superior antiviral functions as increased HIV-specific CTL-stimulation and reduced transfer to CD4^+^ T cells. These effects were likely a consequence of higher viral fusion of HIV-C during co-infection compared to LPS-DCs and the sequential infection model, where DCs were incubated with *Chlamydia* for a prolonged period prior to addition of HIV-C.

Siglec-1 was recently described to play a major role during HIV-1 capture and transfer in LPS-mDCs. Here, we also analyzed co-localization of GFP-tagged complement-opsonized HIV-1 and Siglec-1 in iDCs, HIV+Chlam-, Chlam-, and LPS-DCs. We did not find any correlation between co-localization of Siglec-1/HIV-C, the maturation status of DCs and transfer to susceptible T cells. These findings are consistent with recent *in vivo* studies by Martinez-Picado et al. where they demonstrated that Siglec-1 protein truncation did not have a measurable impact on HIV-1 acquisition or AIDS outcomes *in vivo* (48). The missing correlation of Siglec-1/HIV-C and transfer from differently matured DCs to target cells which was described *in vitro* for non-opsonized HIV-1 by recent studies (41, 49–51) might rely on the fact that C3 fragments covalently bind to the surface of HIV-1 (52) potentially hampering interactions of Siglec-1 with virus-incorporated host-cell-derived glycosphingolipid GM3. GM3 was shown to allow capture by DCs, monocytes and macrophages *in vitro* (51). In our analyses, we, too, found higher co-localization of non-opsonized HIV with Siglec-1, in particular in the sequential infection model, but also in LPS-mDCs. *In vivo*, HIV-1 was found to be opsonized with complement fragments or specific antibodies in all compartments tested so far (53–57). Therefore, the findings by Martinez-Picado et al. that Siglec-1 protein truncation did not correlate with HIV-1 acquisition or AIDS outcomes *in vivo* could be explained by covalent coating of the virus with C3. C3 bound to the viral particles would mediate interactions with complement receptors 3 and 4 (CR3, CD11b/CD18; CR4, CD11c/CD18) abundantly expressed by immature and mature DCs rather than allowing interactions of GM3 with Siglec-1. We earlier found that the covalently linked cloud of C3 fragments on the viral surface impaired interactions of the HIV envelope glycoproteins with C-type lectins expressed on iDCs (8).

The presented data shows that co- or sequential infection of DCs with *Chlamydia* alters the progression of subsequent HIV-1 infection with implications for HIV-1 processing into peptides for MHC presentation, transfer to target cells via *trans*-infection and CTL responses (58, 59). STIs are an important public health issue and in HIV-positive women, STIs are associated not only with gynecological complications but with increased risk of HIV transmission to HIV-negative partners and newborns (60). We find that infection of DCs with HIV-C (or HIV) and *Chlamydia* are associated with mechanisms but only if added simultaneously. The mechanisms are likely due to simultaneous stimulation of innate immune mechanisms on DCs. One such trigger might be activation of Toll-like receptors (TLRs), since *Chlamydia* was illustrated to activate TLR2/6 (61). Therefore, within the chlamydial/HIV-C co-infection model TLRs in concerted action with CR3/CR4 (HIV-C) or C-type lectins (HIV) could stimulate a more robust DC activation compared to HIV-C- or HIV-DCs alone. This would result in even higher stimulation of HIV-specific CTLs and reduction of viral infectivity in the co-infection model. Other host innate immune responses, which might contribute to the higher anti-HIV-1 activity of co-infected DCs comprise superior induction of pro-inflammatory cytokines and/or antimicrobial peptides (62–64). However, the sequential infection model, which probably occurs more often *in vivo* compared to simultaneous DC stimulation with both pathogens, was associated with harm to the host due to significantly enhanced *cis* and *trans* infection with HIV-1 and significantly reduced HIV-specific CTL-stimulation. In future studies, we want to elucidate the mechanisms in DCs involved in the observed differences in *Chlamydia*-mediated effects to characterize factors associated with protection, which might be applied as therapeutic interventions during STIs to lower the risk of HIV-1 transmission and infection.

## Ethics Statement

This study was carried out in accordance with the recommendations of the Ethics Committee of the Medical University of Innsbruck. The protocol was approved by the Ethics Committee of the Medical University of Innsbruck [ECS 1166/2018].

## Author Contributions

MS, UK, and PC performed experiments, analyzed data, and read the manuscript, PH, TJH, AM, and RB-W contributed essential components, read and discussed the manuscript, CL-F helped in designing the study, provided financial support and read and discussed the manuscript, WP and DW designed the study, conducted experiments, analyzed data, and wrote the manuscript with input from all authors. All authors provided critical feedback and helped shape the research, analysis and manuscript.

### Conflict of Interest Statement

The authors declare that the research was conducted in the absence of any commercial or financial relationships that could be construed as a potential conflict of interest.

## Supplementary Material

Figure S1 Flow cytometric analyses of DC profiles of iDCs and differentially treated DCs. (Upper panel) Monocyte-derived iDCs are routinely checked for characteristic markers CD11b, CD11c, and DC-SIGN, which are homogenously expressed on day 5 iDCs. Characteristic maturation markers CD83 and HLA-DR are not expressed or do show a low expression on day 5 iDCs dependent on the donor. Representative histogram plots for the various markers are illustrated. (Lower panel) Day 5 iDCs were treated for further 2 days with live (light green) or heat-inactivated (dark green) Chlamydia or not (iDCs, red) and analyzed for expression of characteristic maturation markers. A representative histogram plot for CD83 is depicted.

Figure S2 Chlam- and LPS-DCs efficiently capture HIV. Binding at 4°C **(A)** and internalization at 37°C **(B)** were performed in triplicates using 25 ng/ml of R5-tropic non-opsonized HIV-1. Bar graphs show means ± SD from three independent experiments. p24 levels within the cell lysates were determined by ELISA. Prior to cell lysate preparation, cells were thoroughly washed to remove unbound virus. Statistical analysis shows 2-way ANOVA with Tukey's multiple comparisons test. Six donors are summarized. **(C)** Fusion assays were performed by exposure of HIV/Chlam-DCs and LPS-DCs to HIV bearing the chimeric protein β-lactamase-vpr. The amount of fused virus was determined by flow cytometric analyses of cleaved CCF2 in the cytoplasm. Percentages of cleaved CCF2-positive cells from three independent donors are depicted.

Figure S3 Siglec-1-independent transfer of HIV-C. Enhanced transfer of HIV-C from Chlam- and LPS-DCs was independent on Siglec-1 as analyzed by high content screening as depicted (upper panel). Only low spots of HIV-C/Siglec-1-co-localization were quantified in 2 fields of 100 cells each (lower panel, right). The co-localization was compared to non-infected differentially stimulated DCs, which represent background values (lower panel, left), and HIV-infected differentially stimulated DCs (lower panel, middle). 200 cells were analyzed in total.

Figure S4 Localization of HIV-C in iDCs and LPS-DCs. For three-dimensional reconstructions, confocal z stacks of iDCs and LPS-DCs exposed to HIV-C were processed with Imaris software using surface reconstruction (Surpass, IMARIS 8.2). About 30 cells per condition were analyzed.

Figure S5 Enhanced DC infection by HIV-C independent of stimulation. iDCs, HIV/Chlam- and Chlam-DCs exerted a significantly enhanced infection using HIV-C (gray) compared to HIV (white). Nevertheless, also productive DC infection of HIV/Chlam-DCs was significantly increased compared to the low-level infection of iDCs using non-opsonized HIV. Three independent donors were summarized in the graph and means ± SD are shown.

Figure S6 Enhanced CTL stimulation by HIV+Chlam DCs. IFNγ induction in CD8+ T cell clones by DCs simultaneously exposed to HIV and Chlamydia was significantly higher than that iDCs, Chlam-, and LPS-DCs exposed to HIV (*p* < 0.0001 for all). Means ± SD of three independent experiments are illustrated.

## References

[1] Doncel GF Joseph T Thurman AR Role of semen in HIV-1 transmission: inhibitor or facilitator? Am J Reproduc Immunol. (2011) 65:292–301. 10.1111/j.1600-0897.2010.00931.x 21087339

[2] Royce RA Seña A Cates WJ Cohen MS. Sexual Transmission of HIV. N Engl J Med. (1997) 336:1072–8. 10.1056/NEJM199704103361507 9091805

[3] Shen R Richter HE Smith PD. Early HIV-1 target cells in human vaginal and ectocervical mucosa. Am J Reproduc Immunol. (2011) 65:261–7. 10.1111/j.1600-0897.2010.00939.x 21118402 PMC3077123

[4] Ebenbichler CF Thielens NM Vornhagen R Marschang P Arlaud GJ Dierich MP. Human immunodeficiency virus type 1 activates the classical pathway of complement by direct C1 binding through specific sites in the transmembrane glycoprotein gp41. J Exp Med. (1991) 174:1417–24. 10.1084/jem.174.6.1417 1744579 PMC2119058

[5] Bouhlal H Chomont N Haeffner-Cavaillon N Kazatchkine MD Belec L Hocini H. Opsonization of HIV-1 by semen complement enhances infection of human epithelial cells. J Immunol. (2002) 169:3301–6. 10.4049/jimmunol.169.6.3301 12218150

[6] Stoiber H Soederholm A Wilflingseder D Gusenbauer S Hildgartner A Dierich MP. Complement and antibodies: a dangerous liaison in HIV infection? Vaccine. 26(Suppl. 8):I79–85. 10.1016/j.vaccine.2008.11.050 19388170

[7] Wilflingseder D Banki Z Garcia E Pruenster M Pfister G Muellauer B. IgG opsonization of HIV impedes provirus formation in and infection of dendritic cells and subsequent long-term transfer to T cells. J Immunol. (2007) 178:7840–8. 10.4049/jimmunol.178.12.7840 17548622

[8] Pruenster M Wilflingseder D Bánki Z Ammann CG Muellauer B Meyer M. C-type lectin-independent interaction of complement opsonized HIV with monocyte-derived dendritic cells. Eur J Immunol. (2005) 35:2691–8. 10.1002/eji.200425940 16094691

[9] Posch W Steger M Knackmuss U Blatzer M Baldauf HM Doppler W. Complement-opsonized HIV-1 overcomes restriction in dendritic cells. PLoS Pathog. (2015) 11:e1005005. 10.1371/journal.ppat.1005005 26121641 PMC4485899

[10] Galvin SR Cohen MS. The role of sexually transmitted diseases in HIV transmission. Nat Rev Microbiol. (2004) 2:33–42. 10.1038/nrmicro794 15035007

[11] Fleming DT Wasserheit JN. From epidemiological synergy to public health policy and practice: the contribution of other sexually transmitted diseases to sexual transmission of HIV infection. Sexually Transmitted Infect. (1999) 75:3–17. 10.1136/sti.75.1.3 10448335 PMC1758168

[12] Hillier SL. Diagnostic microbiology of bacterial vaginosis. Am J Obstetr Gynecol. (1993) 169(2, Part 2):455–9. 10.1016/0002-9378(93)90340-O 8357044

[13] Sewankambo N Gray RH Wawer MJ Paxton L McNairn D Wabwire-Mangen F HIV-1 infection associated with abnormal vaginal flora morphology and bacterial vaginosis. Lancet. (1997) 350:546–50. 10.1016/S0140-6736(97)01063-5 9284776

[14] St John EP, Martinson J, Simoes JA, Landay AL, Spear GT. Dendritic cell activation and maturation induced by mucosal fluid from women with bacterial vaginosis. Clin Immunol. (2007) 125:95–102. 10.1016/j.clim.2007.06.004 17652029 PMC2040390

[15] Bachmann NL Polkinghorne A Timms P. Chlamydia genomics: providing novel insights into chlamydial biology. Trends Microbiol. (2014) 22:464–72. 10.1016/j.tim.2014.04.013 24882432

[16] Banki Z Posch W Ejaz A Oberhauser V Willey S Gassner C. Complement as an endogenous adjuvant for dendritic cell-mediated induction of retrovirus-specific CTLs. PLoS Pathog. (2010) 6:e1000891. 10.1371/journal.ppat.1000891 20442876 PMC2861708

[17] Wilflingseder D Schroll A Hackl H Gallasch R Frampton D Lass-Florl C. Immediate T-helper 17 polarization upon triggering CD11b/c on HIV-exposed dendritic cells. J Infect Dis. (2015) 212:44–56. 10.1093/infdis/jiv014 25583169

[18] Sullivan BL Knopoff EJ Saifuddin M Takefman DM Saarloos MN Sha BE. Susceptibility of HIV-1 Plasma Virus to Complement-mediated lysis. Immunol J. (1996) 157:1791–8. 8759769

[19] Posch W Cardinaud S Hamimi C Fletcher A Muhlbacher A Loacker K. Antibodies attenuate the capacity of dendritic cells to stimulate HIV-specific cytotoxic T lymphocytes. J Allergy Clin Immunol. (2012) 130:1368–74 e2. 10.1016/j.jaci.2012.08.025 23063584 PMC4834990

[20] Bellmann-Weiler R Martinz V Kurz K Engl S Feistritzer C Fuchs D. Divergent modulation of Chlamydia pneumoniae infection cycle in human monocytic and endothelial cells by iron, tryptophan availability and interferon gamma. Immunobiology. (2010) 215:842–8. 10.1016/j.imbio.2010.05.021 20646782

[21] Campbell EM Perez O Anderson JL Hope TJ. Visualization of a proteasome-independent intermediate during restriction of HIV-1 by rhesus TRIM5alpha. J Cell Biol. (2008) 180:549–61. 10.1083/jcb.200706154 18250195 PMC2234241

[22] Purtscher M Trkola A Gruber G Buchacher A Predl R Steindl F. A broadly neutralizing human monoclonal antibody against gp41 of human immunodeficiency virus type 1. AIDS Res Hum Retroviruses. (1994) 10:1651–8. 10.1089/aid.1994.10.1651 7888224

[23] Moris A Nobile C Buseyne F Porrot F Abastado JP Schwartz O. DC-SIGN promotes exogenous MHC-I-restricted HIV-1 antigen presentation. Blood. (2004) 103:2648–54. 10.1182/blood-2003-07-2532 14576049

[24] Casartelli N Guivel-Benhassine F Bouziat R Brandler S Schwartz O Moris A. The antiviral factor APOBEC3G improves CTL recognition of cultured HIV-infected T cells. J Exp Med. (2010) 207:39–49. 10.1084/jem.20091933 20038599 PMC2812543

[25] Ogg GS Jin X Bonhoeffer S Dunbar PR Nowak MA Monard S. Quantitation of HIV-1-specific cytotoxic T lymphocytes and plasma load of viral RNA. Science. (1998) 279:2103–6. 10.1126/science.279.5359.2103 9516110

[26] Chen Q Swaminathan S Yang D Dai L Sui H Yang J. Interleukin-27 is a potent inhibitor of cis HIV-1 replication in monocyte-derived dendritic cells via a type I interferon-independent pathway. PLoS ONE. (2013) 8:e59194. 10.1371/journal.pone.0059194 23527130 PMC3604098

[27] Rodriguez-Plata MT Puigdomenech I Izquierdo-Useros N Puertas MC Carrillo J Erkizia I. The infectious synapse formed between mature dendritic cells and CD4(+) T cells is independent of the presence of the HIV-1 envelope glycoprotein. Retrovirology. (2013) 10:42. 10.1186/1742-4690-10-42 23590845 PMC3640963

[28] Izquierdo-Useros N Lorizate M Puertas MC Rodriguez-Plata MT Zangger N Erikson E. Siglec-1 is a novel dendritic cell receptor that mediates HIV-1 trans-infection through recognition of viral membrane gangliosides. PLoS Biol. (2012) 10:e1001448. 10.1371/journal.pbio.1001448 23271952 PMC3525531

[29] Martinez-Picado J McLaren PJ Telenti A Izquierdo-Useros N. Retroviruses as myeloid cell riders: what natural human siglec-1 “Knockouts” tell us about pathogenesis. Front Immunol. (2017) 8:1593. 10.3389/fimmu.2017.01593 29209326 PMC5702442

[30] Izquierdo-Useros N Lorizate M McLaren PJ Telenti A Krausslich HG Martinez-Picado J. HIV-1 capture and transmission by dendritic cells: the role of viral glycolipids and the cellular receptor Siglec-1. PLoS Pathog. (2014) 10:e1004146. 10.1371/journal.ppat.1004146 25033082 PMC4102576

[31] Izquierdo-Useros N Naranjo-Gomez M Erkizia I Puertas MC Borras FE Blanco J. HIV and mature dendritic cells: trojan exosomes riding the Trojan horse? PLoS Pathog. (2010) 6:e1000740. 10.1371/journal.ppat.1000740 20360840 PMC2845607

[32] Slade J Hall JV Kintner J Schoborg RV. Chlamydial pre-infection protects from subsequent herpes simplex virus-2 challenge in a murine vaginal super-infection model. PLoS ONE. (2016) 11:e0146186. 10.1371/journal.pone.0146186 26726882 PMC4699815

[33] Sheung A Rebbapragada A Shin LY Dobson-Belaire W Kimani J Ngugi E. Mucosal Neisseria gonorrhoeae coinfection during HIV acquisition is associated with enhanced systemic HIV-specific CD8 T-cell responses. AIDS. (2008) 22:1729–37. 10.1097/QAD.0b013e32830baf5e 18753933

[34] Cavrois M Neidleman J Kreisberg JF Fenard D Callebaut C Greene WC. Human immunodeficiency virus fusion to dendritic cells declines as cells mature. J Virol. (2006) 80:1992–9. 10.1128/JVI.80.4.1992-1999.2006 16439555 PMC1367165

[35] Deneka M Pelchen-Matthews A Byland R Ruiz-Mateos E Marsh M. In macrophages, HIV-1 assembles into an intracellular plasma membrane domain containing the tetraspanins CD81, CD9, and CD53. J Cell Biol. (2007) 177:329–41. 10.1083/jcb.200609050 17438075 PMC2064140

[36] Pelchen-Matthews A Kramer B Marsh M. Infectious HIV-1 assembles in late endosomes in primary macrophages. J Cell Biol. (2003) 162:443–55. 10.1083/jcb.200304008 12885763 PMC2172706

[37] Bennett AE Narayan K Shi D Hartnell LM Gousset K He H. Ion-abrasion scanning electron microscopy reveals surface-connected tubular conduits in HIV-infected macrophages. PLoS Pathog. (2009) 5:e1000591. 10.1371/journal.ppat.1000591 19779568 PMC2743285

[38] Welsch S Keppler OT Habermann A Allespach I Krijnse-Locker J Krausslich HG. HIV-1 buds predominantly at the plasma membrane of primary human macrophages. PLoS Pathog. (2007) 3:e36. 10.1371/journal.ppat.0030036 17381240 PMC1829407

[39] Hammonds JE Beeman N Ding L Takushi S Francis AC Wang JJ. Siglec-1 initiates formation of the virus-containing compartment and enhances macrophage-to-T cell transmission of HIV-1. PLoS Pathog. (2017) 13:e1006181. 10.1371/journal.ppat.1006181 28129379 PMC5298340

[40] Puryear WB Akiyama H Geer SD Ramirez NP Yu X Reinhard BM. Interferon-inducible mechanism of dendritic cell-mediated HIV-1 dissemination is dependent on Siglec-1/CD169. PLoS Pathog. (2013) 9:e1003291. 10.1371/journal.ppat.1003291 23593001 PMC3623718

[41] Puryear WB Gummuluru S. Role of glycosphingolipids in dendritic cell-mediated HIV-1 trans-infection. Adv Exp Med Biol. (2013) 762:131–53. 10.1007/978-1-4614-4433-6_5 22975874 PMC3686569

[42] Yu HJ Reuter MA McDonald D. HIV traffics through a specialized, surface-accessible intracellular compartment during trans-infection of T cells by mature dendritic cells. PLoS Pathog. (2008) 4:e1000134. 10.1371/journal.ppat.1000134 18725936 PMC2515344

[43] Garcia E Pion M Pelchen-Matthews A Collinson L Arrighi JF Blot G. HIV-1 trafficking to the dendritic cell-T-cell infectious synapse uses a pathway of tetraspanin sorting to the immunological synapse. Traffic. (2005) 6:488–501. 10.1111/j.1600-0854.2005.00293.x 15882445

[44] Izquierdo-Useros N Esteban O Rodriguez-Plata MT Erkizia I Prado JG Blanco J. Dynamic imaging of cell-free and cell-associated viral capture in mature dendritic cells. Traffic. (2011) 12:1702–13. 10.1111/j.1600-0854.2011.01281.x 21917091

[45] Sanders RW de Jong EC Baldwin CE Schuitemaker JH Kapsenberg ML Berkhout B. Differential transmission of human immunodeficiency virus type 1 by distinct subsets of effector dendritic cells. J Virol. (2002) 76:7812–21. 10.1128/JVI.76.15.7812-7821.2002 12097593 PMC136398

[46] Wang JH Janas AM Olson WJ Wu L. Functionally distinct transmission of human immunodeficiency virus type 1 mediated by immature and mature dendritic cells. J Virol. (2007) 81:8933–43. 10.1128/JVI.00878-07 17567699 PMC1951429

[47] Granelli-Piperno A Delgado E Finkel V Paxton W Steinman RM. Immature dendritic cells selectively replicate macrophagetropic (M-tropic) human immunodeficiency virus type 1, while mature cells efficiently transmit both M- and T-tropic virus to T cells. J Virol. (1998) 72:2733–7. 9525591 10.1128/jvi.72.4.2733-2737.1998 PMC109716

[48] Martinez-Picado J McLaren PJ Erkizia I Martin MP Benet S Rotger M. Identification of Siglec-1 null individuals infected with HIV-1. Nat Commun. (2016) 7:12412. 10.1038/ncomms12412 27510803 PMC4987525

[49] Pino M Erkizia I Benet S Erikson E Fernandez-Figueras MT Guerrero D Dalmau J. HIV-1 immune activation induces Siglec-1 expression and enhances viral trans-infection in blood and tissue myeloid cells. Retrovirology. (2015) 12:37. 10.1186/s12977-015-0160-x 25947229 PMC4423124

[50] Izquierdo-Useros N Naranjo-Gomez M Archer J Hatch SC Erkizia I Blanco J. Capture and transfer of HIV-1 particles by mature dendritic cells converges with the exosome-dissemination pathway. Blood. (2009) 113:2732–41. 10.1182/blood-2008-05-158642 18945959 PMC2661860

[51] Puryear WB Yu X Ramirez NP Reinhard BM Gummuluru S. HIV-1 incorporation of host-cell-derived glycosphingolipid GM3 allows for capture by mature dendritic cells. Proc Natl Acad Sci USA. (2012) 109:7475–80. 10.1073/pnas.1201104109 22529395 PMC3358844

[52] Stoiber H Ebenbichler C Schneider R Janatova J Dierich MP. Interaction of several complement proteins with gp120 and gp41, the two envelope glycoproteins of HIV-1. AIDS. (1995) 9:19–26. 10.1097/00002030-199501000-00003 7893437

[53] Hess C Klimkait T Schlapbach L Del Zenero V Sadallah S Horakova E. Association of a pool of HIV-1 with erythrocytes *in vivo*: a cohort study. Lancet. (2002) 359:2230–4. 10.1016/S0140-6736(02)09291-7 12103286

[54] Burton GF Masuda A Heath SL Smith BA Tew JG Szakal AK. Follicular dendritic cells (FDC) in retroviral infection: host/pathogen perspectives. Immunol Rev. (1997) 156:185–97. 10.1111/j.1600-065X.1997.tb00968.x 9176708

[55] Moir S Malaspina A Li Y Chun TW Lowe T Adelsberger J. B cells of HIV-1-infected patients bind virions through CD21-complement interactions and transmit infectious virus to activated T cells. J Exp Med. (2000) 192:637–46. 10.1084/jem.192.5.637 10974030 PMC2193277

[56] Kacani L Prodinger WM Sprinzl GM Schwendinger MG Spruth M Stoiber H. Detachment of human immunodeficiency virus type 1 from germinal centers by blocking complement receptor type 2. J Virol. (2000) 74:7997–8002. 10.1128/JVI.74.17.7997-8002.2000 10933708 PMC112331

[57] Schifferli JA. Complement and immune complexes. Res Immunol. (1996) 147:109–10. 10.1016/0923-2494(96)87183-5 8792470

[58] McDonald D Wu L Bohks SM KewalRamani VN Unutmaz D Hope TJ. Recruitment of HIV and its receptors to dendritic cell-T cell junctions. Science. (2003) 300:1295–7. 10.1126/science.1084238 12730499

[59] Turville SG Santos JJ Frank I Cameron PU Wilkinson J Miranda-Saksena M. Immunodeficiency virus uptake, turnover, and 2-phase transfer in human dendritic cells. Blood. (2004) 103:2170–9. 10.1182/blood-2003-09-3129 14630806

[60] Alcaide ML Jones DL Chitalu N Weiss S. Chlamydia and Gonorrhea Infections in HIV-positive Women in Urban Lusaka, Zambia. J Glob Infect Dis. (2012) 4:141–4. 10.4103/0974-777X.100566 23055644 PMC3459430

[61] Wang Y Liu Q Chen D Guan J Ma L Zhong G. Chlamydial lipoproteins stimulate toll-like receptors 1/2 mediated inflammatory responses through MyD88-dependent pathway. Front Microbiol. (2017) 8:78. 10.3389/fmicb.2017.00078 28184217 PMC5266682

[62] Liechti GW Kuru E Hall E Kalinda A Brun YV VanNieuwenhze M. A new metabolic cell-wall labelling method reveals peptidoglycan in Chlamydia trachomatis. Nature. (2014) 506:507–10. 10.1038/nature12892 24336210 PMC3997218

[63] Hickey DK Patel MV Fahey JV Wira CR. Innate and adaptive immunity at mucosal surfaces of the female reproductive tract: stratification and integration of immune protection against the transmission of sexually transmitted infections. J Reprod Immunol. (2011) 88:185–94. 10.1016/j.jri.2011.01.005 21353708 PMC3094911

[64] Wira CR Patel MV Ghosh M Mukura L Fahey JV. Innate immunity in the human female reproductive tract: endocrine regulation of endogenous antimicrobial protection against HIV and other sexually transmitted infections. Am J Reprod Immunol. (2011) 65:196–211. 10.1111/j.1600-0897.2011.00970.x 21294805 PMC3837338

