# Supplementary figures and images for: Co- but not Sequential Infection of DCs Boosts Their HIV-Specific CTL-Stimulatory Capacity
Source: Front Immunol. 2019 May 24;10:1123. doi: 10.3389/fimmu.2019.01123 (PMC6542955; doi:10.3389/fimmu.2019.01123)

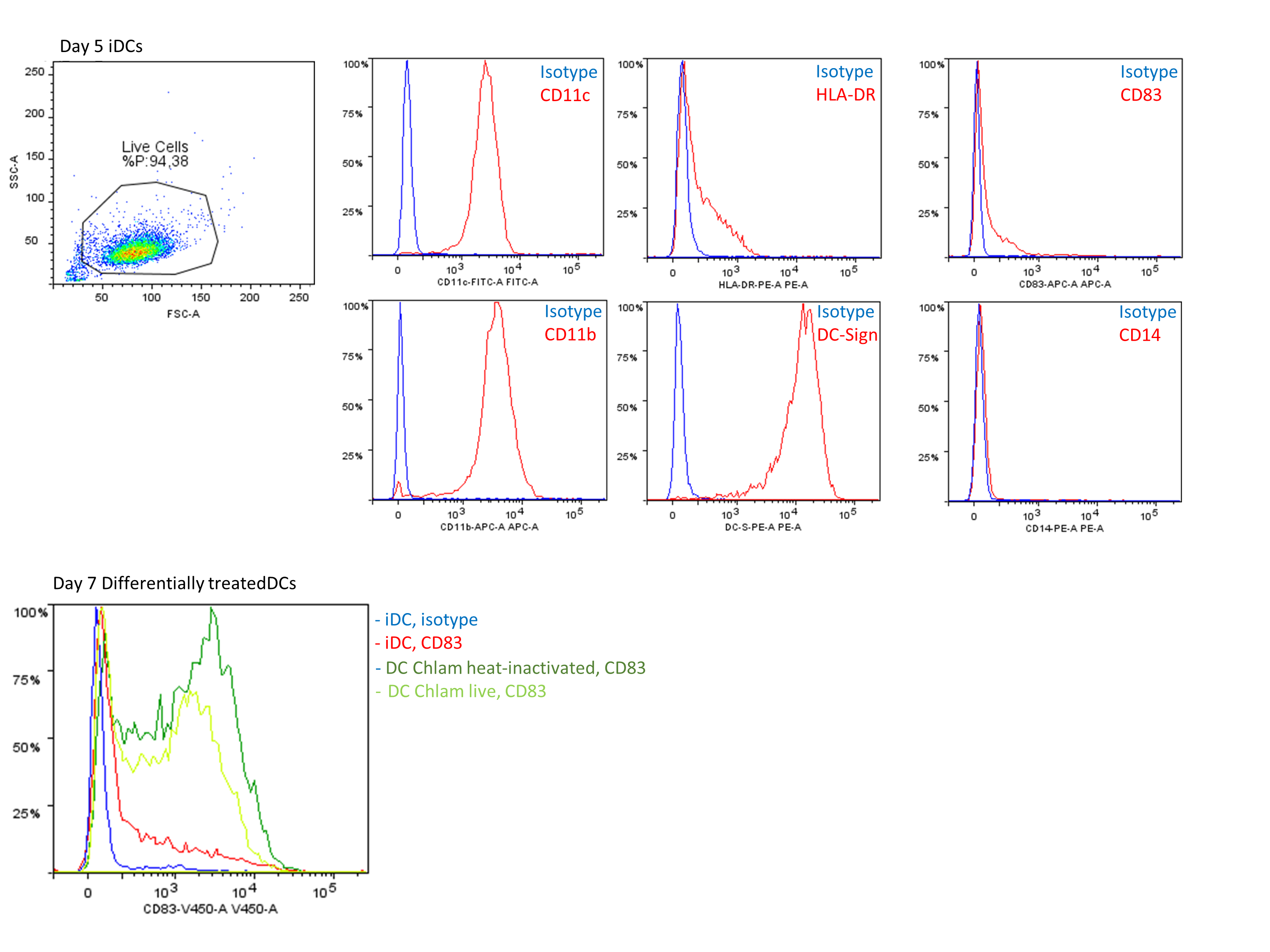

Supplement: Figure S1 — Flow cytometric analyses of DC profiles of iDCs and differentially treated DCs. (Upper panel) Monocyte-derived iDCs are routinely checked for characteristic markers CD11b, CD11c, and DC-SIGN, which are homogenously expressed on day 5 iDCs. Characteristic maturation markers CD83 and HLA-DR are not expressed or do show a low expression on day 5 iDCs dependent on the donor. Representative histogram plots for the various markers are illustrated. (Lower panel) Day 5 iDCs were treated for further 2 days with live (light green) or heat-inactivated (dark green) Chlamydia or not (iDCs, red) and analyzed for expression of characteristic maturation markers. A representative histogram plot for CD83 is depicted. [file Image_1.TIF]

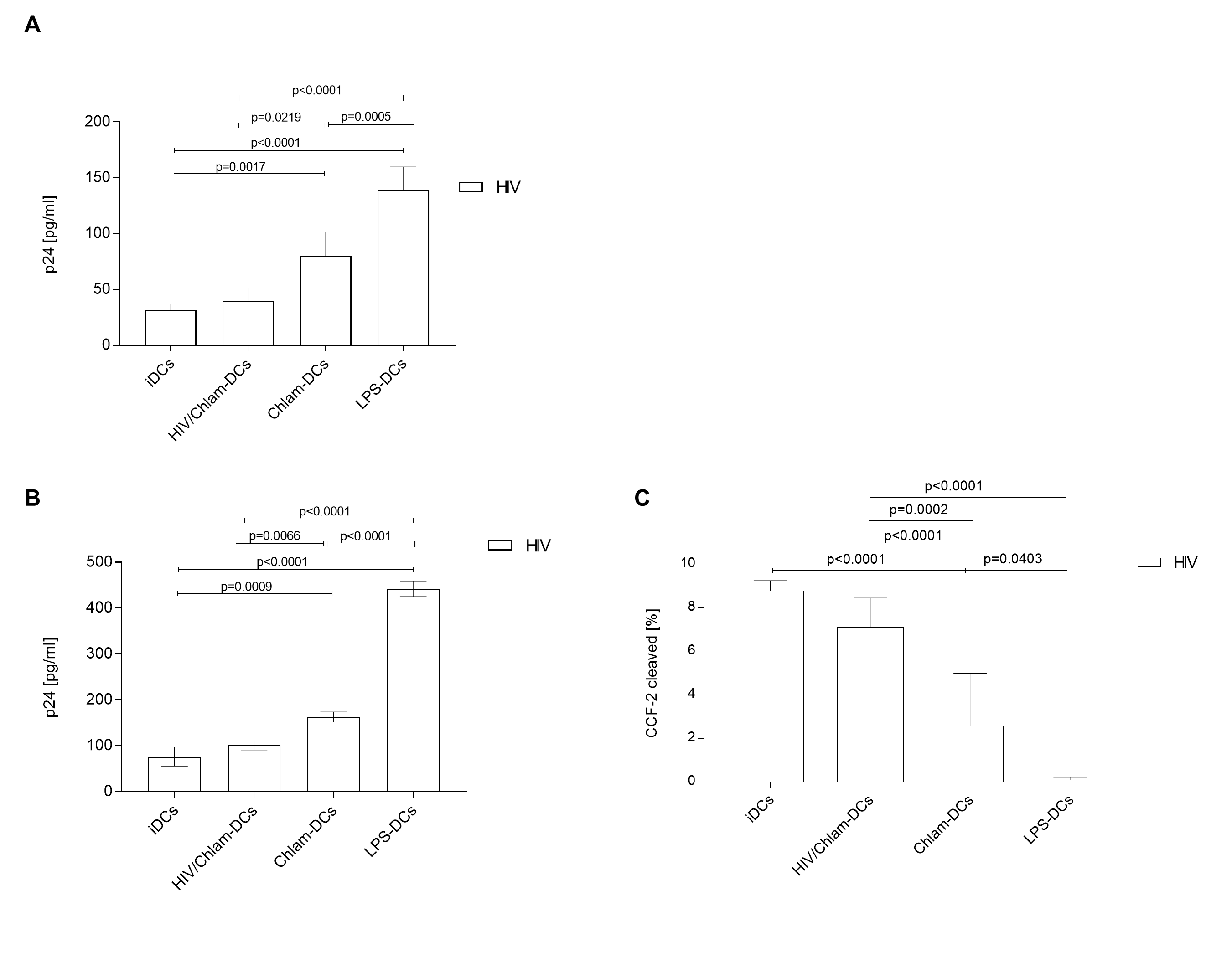

Supplement: Figure S2 — Chlam- and LPS-DCs efficiently capture HIV. Binding at 4°C (A) and internalization at 37°C (B) were performed in triplicates using 25 ng/ml of R5-tropic non-opsonized HIV-1. Bar graphs show means ± SD from three independent experiments. p24 levels within the cell lysates were determined by ELISA. Prior to cell lysate preparation, cells were thoroughly washed to remove unbound virus. Statistical analysis shows 2-way ANOVA with Tukey's multiple comparisons test. Six donors are summarized. (C) Fusion assays were performed by exposure of HIV/Chlam-DCs and LPS-DCs to HIV bearing the chimeric protein β-lactamase-vpr. The amount of fused virus was determined by flow cytometric analyses of cleaved CCF2 in the cytoplasm. Percentages of cleaved CCF2-positive cells from three independent donors are depicted. [file Image_2.TIF]

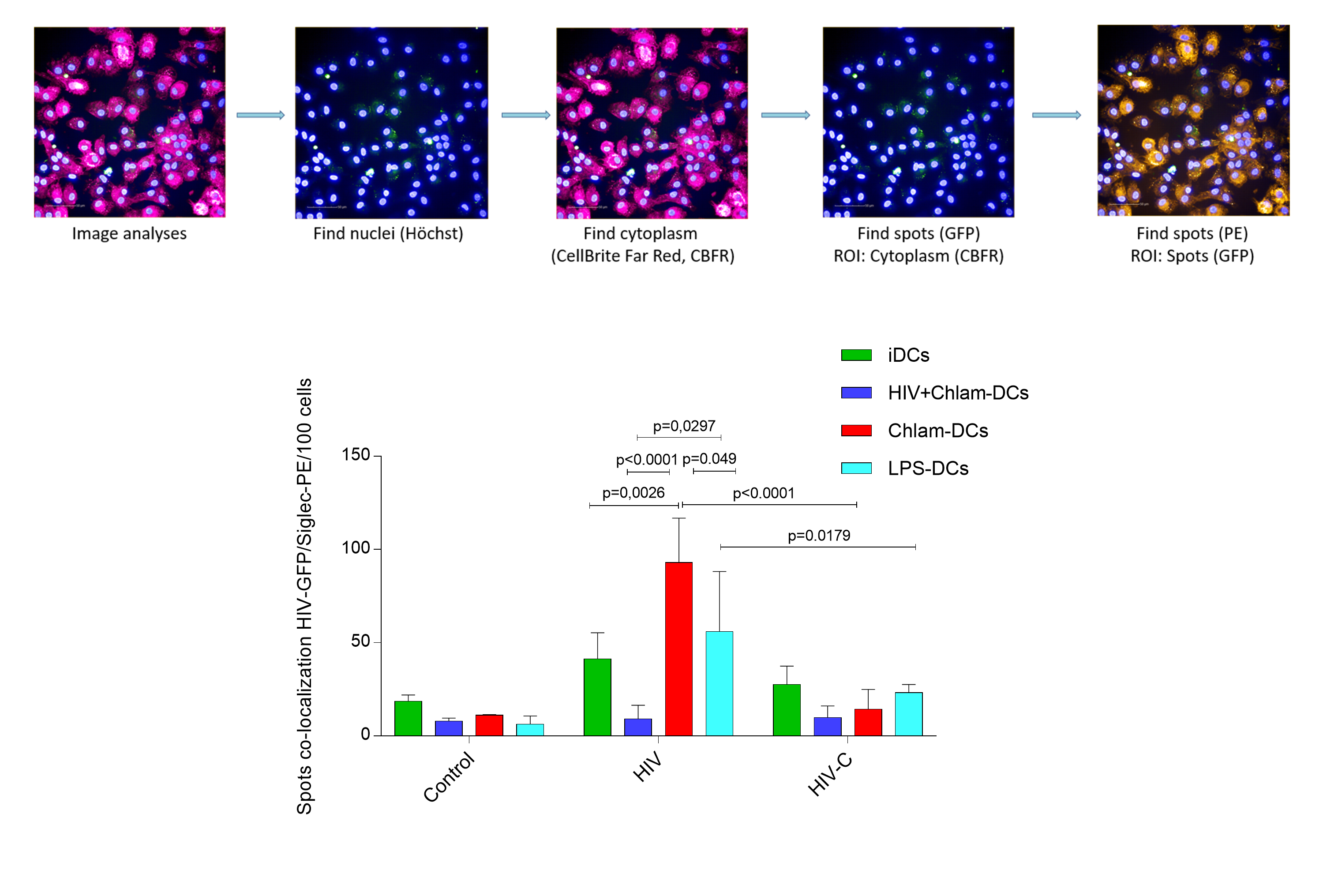

Supplement: Figure S3 — Siglec-1-independent transfer of HIV-C. Enhanced transfer of HIV-C from Chlam- and LPS-DCs was independent on Siglec-1 as analyzed by high content screening as depicted (upper panel). Only low spots of HIV-C/Siglec-1-co-localization were quantified in 2 fields of 100 cells each (lower panel, right). The co-localization was compared to non-infected differentially stimulated DCs, which represent background values (lower panel, left), and HIV-infected differentially stimulated DCs (lower panel, middle). 200 cells were analyzed in total. [file Image_3.TIF]

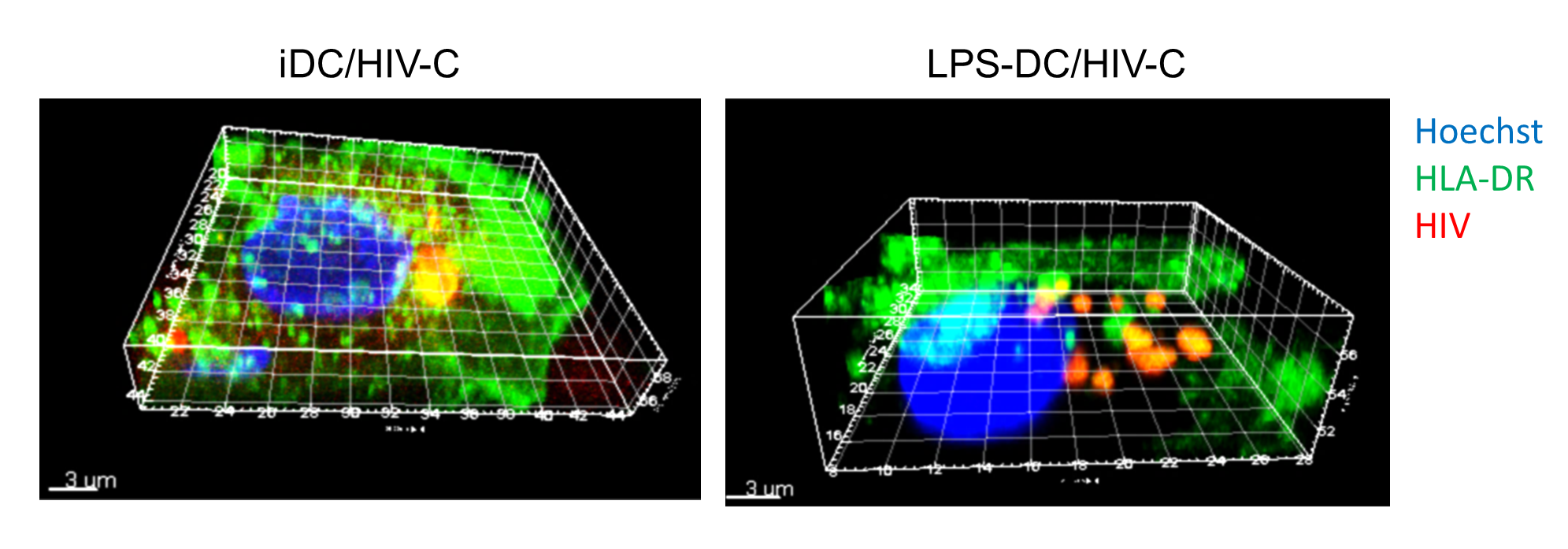

Supplement: Figure S4 — Localization of HIV-C in iDCs and LPS-DCs. For three-dimensional reconstructions, confocal z stacks of iDCs and LPS-DCs exposed to HIV-C were processed with Imaris software using surface reconstruction (Surpass, IMARIS 8.2). About 30 cells per condition were analyzed. [file Image_4.TIF]

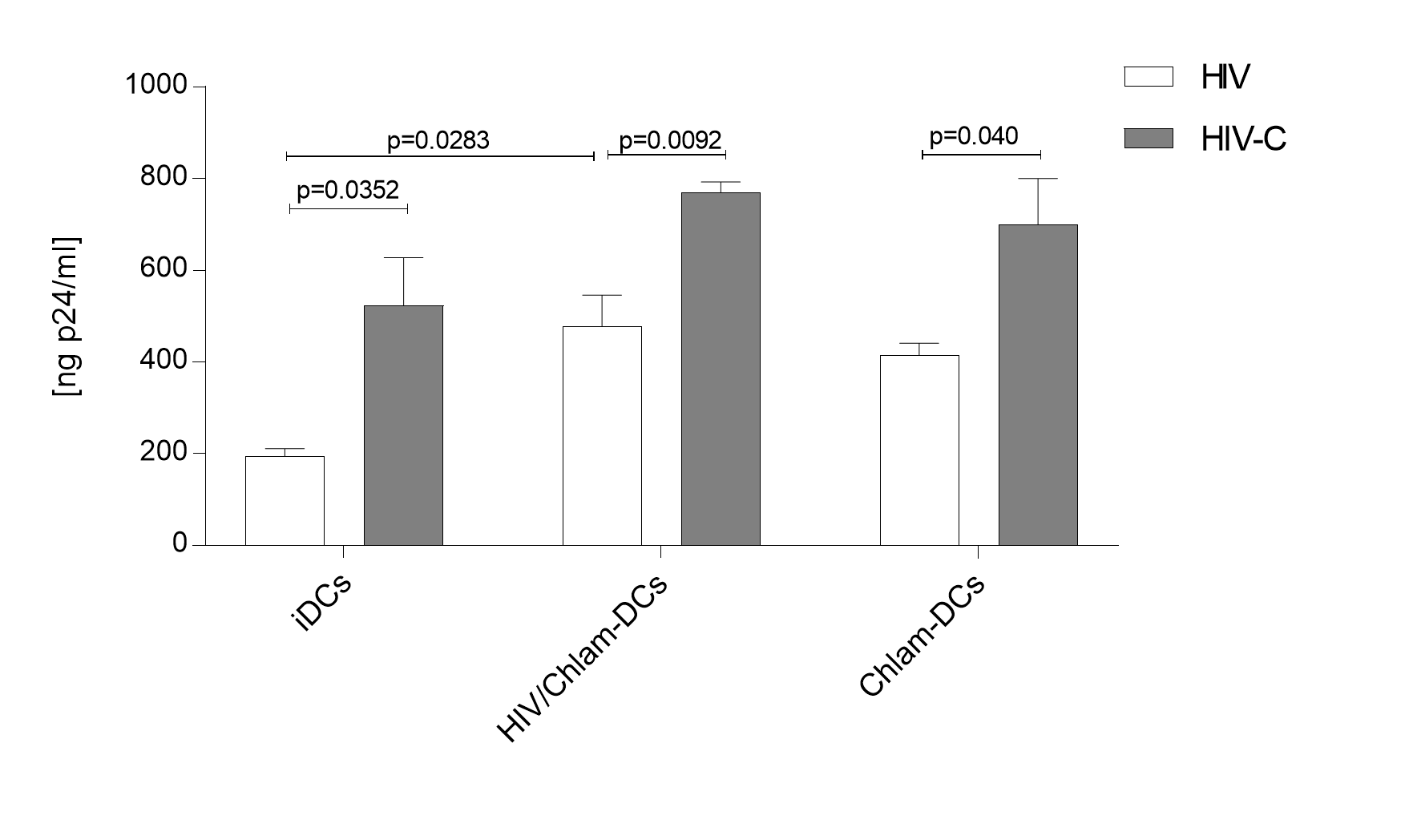

Supplement: Figure S5 — Enhanced DC infection by HIV-C independent of stimulation. iDCs, HIV/Chlam- and Chlam-DCs exerted a significantly enhanced infection using HIV-C (gray) compared to HIV (white). Nevertheless, also productive DC infection of HIV/Chlam-DCs was significantly increased compared to the low-level infection of iDCs using non-opsonized HIV. Three independent donors were summarized in the graph and means ± SD are shown. [file Image_5.TIF]

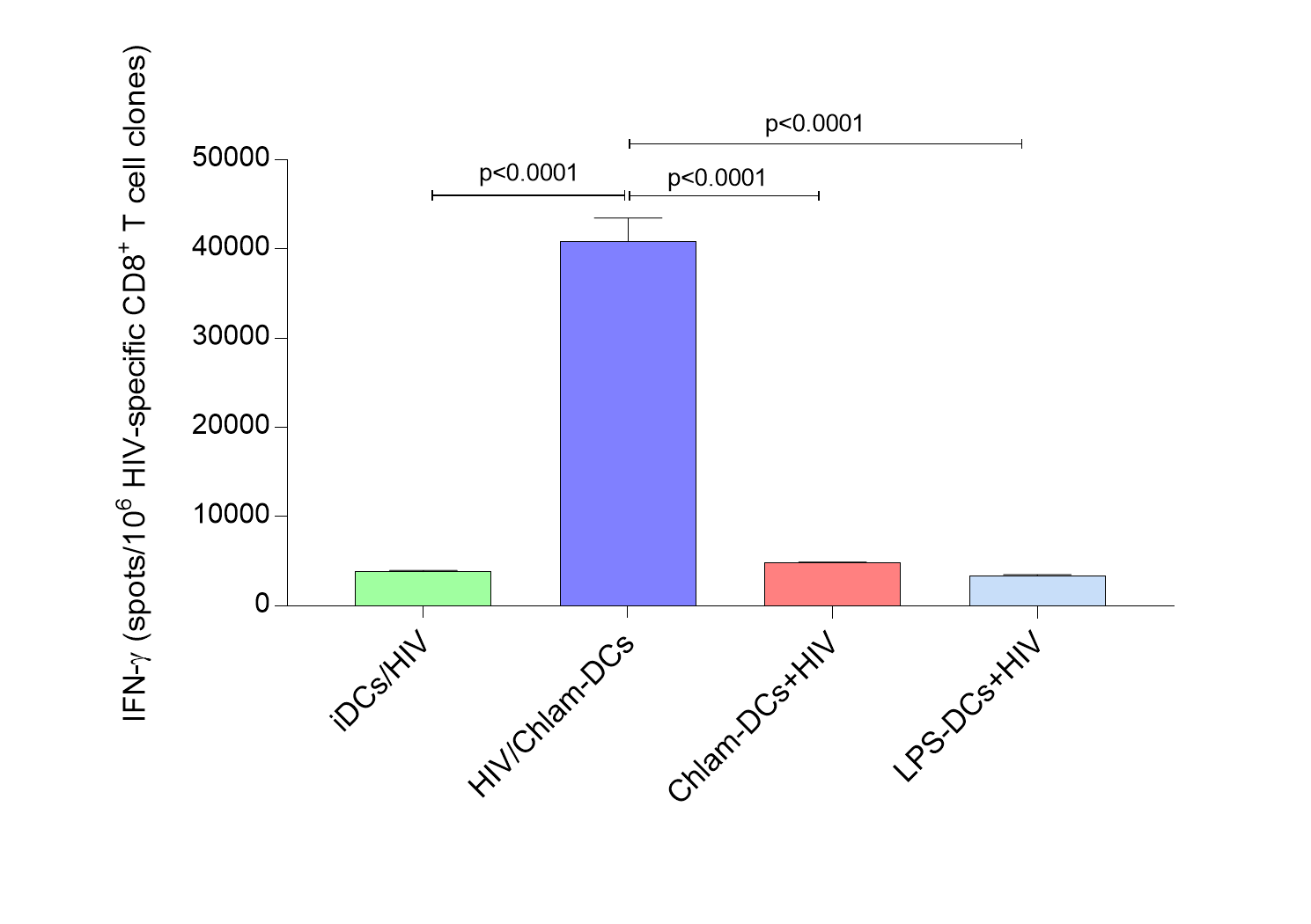

Supplement: Figure S6 — Enhanced CTL stimulation by HIV+Chlam DCs. IFNγ induction in CD8+ T cell clones by DCs simultaneously exposed to HIV and Chlamydia was significantly higher than that iDCs, Chlam-, and LPS-DCs exposed to HIV (p < 0.0001 for all). Means ± SD of three independent experiments are illustrated. [file Image_6.TIF]
